# Mechanisms of intracellular pH gating and local anesthetic inhibition of the two-pore domain K^+^ channel TASK-2

**DOI:** 10.1073/pnas.2604873123

**Published:** 2026-10-08

**Authors:** Trevor Docter, Ben Sorum, Baobin Li, Robert A. Rietmeijer, Annan S. I. Cook, Abhay Kotecha, Stephen G. Brohawn

**Affiliations:** ^a^Department of Molecular and Cell Biology, University of California Berkeley, Berkeley, CA 94720; ^b^Department of Neuroscience, University of California Berkeley, Berkeley, CA 94720; ^c^Helen Wills Neuroscience Institute, University of California Berkeley, Berkeley, CA 94720; ^d^ Biophysics Graduate Program, University of California, Berkeley, CA 94720; ^e^Materials and Structural Analysis, Thermo Fisher Scientific, Eindhoven 5651 GG, The Netherlands; ^f^ California Institute for Quantitative Biosciences, University of California, Berkeley, CA 94720

**Keywords:** pH sensing, K2P channel, local anesthetic, cryo-EM

## Abstract

TWIK-related acid-sensitive K+ channel 2 (TASK-2) is a pH sensing two-pore K^+^ (K2P) channel that regulates respiration in brainstem neurons and systemic pH homeostasis in kidney. Despite its physiological importance and implication in disease, the molecular basis for intracellular gating and pharmacological inhibition of TASK-2 is incompletely understood. Here, we combine cryo-EM and single-channel electrophysiology to resolve the mechanisms of TASK-2 gating by intracellular protons and inhibition by the anesthetic bupivacaine. We show protonation of intracellular lysines triggers a unique stacked gating mechanism distinct from other K^+^ channels. Inner helices unwind and domain swap to form a β-zipper, which we call a Z-gate, that seals the cytoplasmic channel entrance. These conformational changes open lateral fenestrations to the membrane that permit lipids to invade the channel cavity and block the pore. Furthermore, we find bupivacaine inhibits the channel by competing with lipids in the cavity site in a state-dependent manner. These results define a paradigm for intracellular gating of K2Ps and provide a structural foundation for designing more potent and selective channel modulators.

## Significance

The potassium channel TWIK-related acid-sensitive K^+^ channel 2 (TASK-2) is a critical physiological sensor that regulates breathing and kidney function to maintain pH homeostasis. Despite its importance, the molecular mechanisms controlling its intracellular gating and inhibition have remained unclear. We use cryoelectron microscopy and electrophysiology to address these questions. We found TASK-2 employs a unique mechanism to gate closed in response to low intracellular pH that involves protein and lipid components. We further show that the local anesthetic bupivacaine inhibits TASK-2 by binding inside the channel at a site that overlaps with the lipid binding site. These findings define a paradigm for intracellular channel regulation and offer a structural blueprint for designing specific inhibitors of TASK-2.

TWIK-related acid-sensitive K^+^ channel 2 (TASK-2) is a pH-regulated two-pore domain K^+^ (K2P) channel uniquely activated by intracellular and extracellular alkalinization with half-maximal activity at pH_int/ext_ 8 to 8.5 (1–5). TASK-2 is expressed widely, including in central neurons, kidney epithelial cells, pancreatic β cells, immune cells, and chondrocytes (5–9). In brainstem chemosensory neurons of the retrotrapezoid nucleus, TASK-2 senses pH changes as a proxy for *P*CO_2_. Hypercapnic acidification inhibits and hypocapnic alkalosis activates TASK-2, increasing or decreasing spike frequency and respiration rate through input to the pre-Bötzinger complex to maintain *P*CO_2_ homeostasis (10–13). In the kidney proximal tubule, depolarizing electrogenic bicarbonate export results in alkalinization that activates TASK-2, generating a hyperpolarizing current that maintains the outwardly directed driving force required for further bicarbonate efflux (6, 14). Consistently, TASK-2 knockout mice show altered ventilatory response and metabolic acidosis (11, 14). In humans, TASK-2 loss causes a Balkan endemic nephropathy (15) and the channel has been implicated in renal fibrosis (16), migraine (17), ischemic neurodegeneration (18), breast cancer proliferation (19), and dry eye pathogenesis (20).

We previously determined cryo-EM structures of mouse TASK-2 in open (pH 8.5) and closed (pH 6.5) states (21). TASK-2 adopts the canonical K2P fold with each subunit of the dimeric channel composed of four transmembrane helices (TM1–4), an extracellular cap, and two selectivity filter forming regions (SF1 and SF2). External pH gating involves a C-type selectivity filter gate. Protonation of the primary pH_ext_ sensor, R224 at the extracellular end of TM4^2^, results in conformational changes that propagate through the helical cap to disrupt the two outermost K^+^ coordination sites S0 and S1, rendering the selectivity filter nonconductive. A lysine (K245) at the intracellular end of TM4 is the primary proton sensor for intracellular gating of TASK-2^3^. At low pH, conformational changes to the intracellular half of TM4, including K245, create a protein seal at the inner leaflet. These rearrangements also open lateral fenestrations to the membrane large enough to permit lipid access to the channel cavity (21). Lipid block through membrane facing fenestrations contributes to gating in other K2Ps including TWIK and mechanosensitive TRAAK/TREK channels (22–27). However, the contribution of lipids to gating TASK-2 and the precise intracellular gating mechanism are unclear, due in part to the low resolution of the previous TASK-2 structures and the particularly low local resolution of the intracellular side (21).

Despite implication in multiple disease states, pharmacological tools to manipulate TASK-2 are limited. The local anesthetic bupivacaine is the most potent characterized inhibitor; its effects on TASK-2 may contribute to its respiratory and cardiac side effects in addition to nerve block (28). Bupivacaine inhibits voltage-gated sodium channels by binding below the inner bundle crossing gate formed between the four S6 helices (site BIG) (29). However, it must bind differently to dimeric TASK-2, which cannot form an analogous inner bundle crossing binding site. Molecular dynamics simulations suggest a binding site in the lateral fenestrations of TASK-2 and TASK-1 channels (30, 31), but the structural basis for bupivacaine inhibition of TASK-2 has not been directly demonstrated.

Here, using cryo-EM and electrophysiology, we show that TASK-2 intracellular gating involves two interdependent stacked modules. At the base of the channel, a Z (zipper)-gate formed by the inner halves of TM4s creates a protein seal to the cytoplasm. Lipid entry into the channel cavity between the Z-gate and the selectivity filter blocks the conduction path. Mutant analysis shows that abrogation of pH_int_-gating requires disruption of both the Z-gate and lipid block. We further demonstrate that bupivacaine and lipid acyl chains bind in overlapping cavity sites and propose they similarly inhibit ion flux through direct pore block.

## Results

### Cryo-EM Structure of TASK-2_WT_ at pH 6.5.

**Fig. 1.**
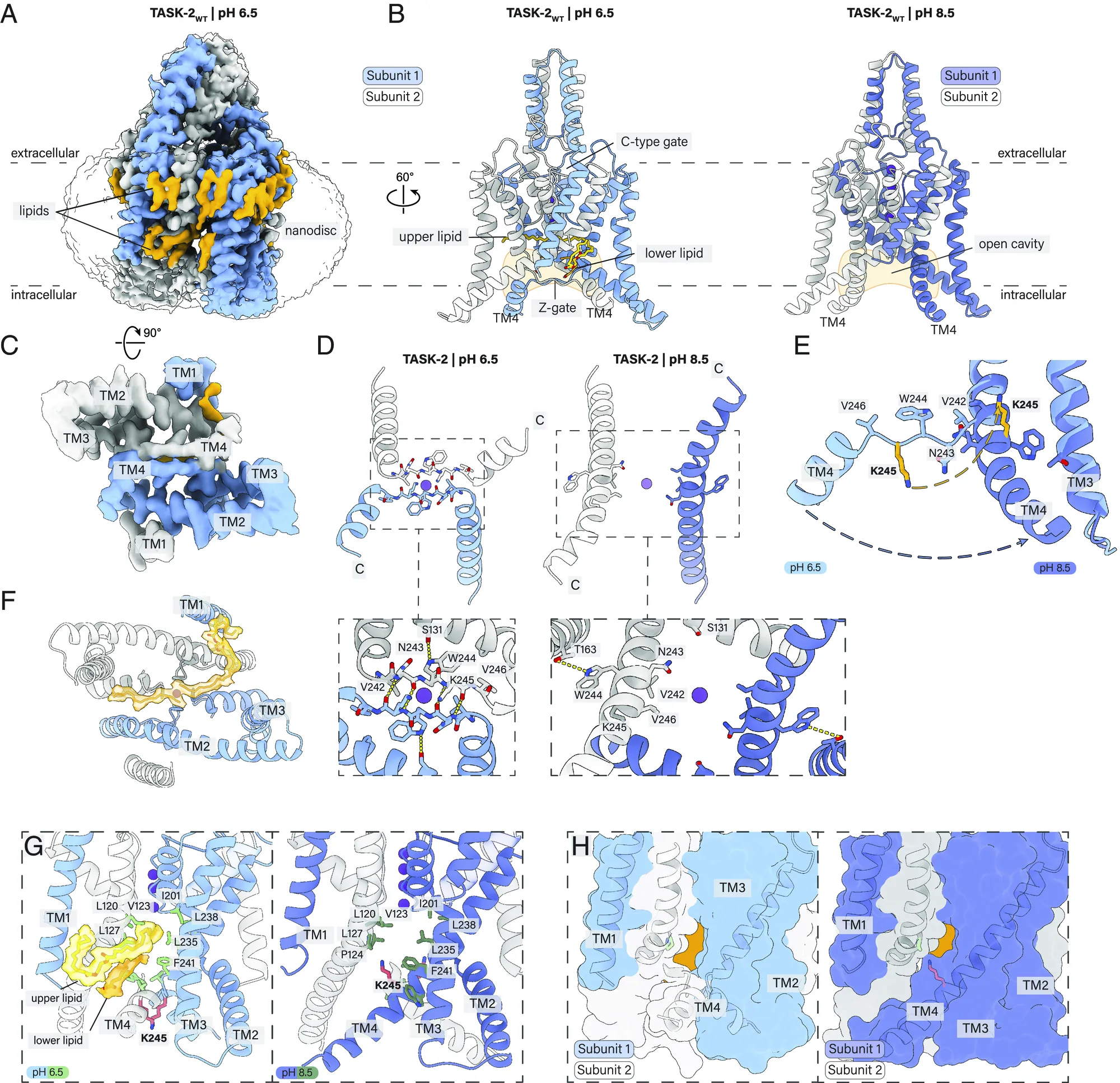
Structure of TASK-2_WT_ at pH 6.5 reveals an intracellular Z-gate and lipid block of the pore. (*A*) Side view of TASK-2_WT_ pH 6.5 cryo-EM map, (*B*) pH 6.5 closed channel model and pH 8.5 open channel model (PDB: 6WM0). Channel subunits are blue and white, K^+^ is purple, and lipids are gold. (*C*) Cytosolic view of TASK-2_WT_ pH 6.5 unsharpened cryo-EM map and (*D*) TM4 configuration at pH 6.5 (*Left*) and pH 8.5 (*Right*) with zoomed views of boxed regions (below). (*E*) Side view of one TM4 at pH 6.5 and pH 8.5 overlaid. (*F*) Cytosolic view of TASK-2_WT_ at pH 6.5 with cavity lipids and carved cryo-EM density. (*G*) Side views of pH 6.5 (*Left*) and pH 8.5 (*Right*) models and (*H*) surfaces highlighting cavity environment and lateral fenestration (orange).

To better understand the mechanisms of intracellular gating in TASK-2, we determined a cryo-EM structure of mouse TASK-2 (subsequently referred to as TASK-2_WT_) in lipid nanodiscs at pH 6.5 to an overall resolution of 2.6 Å (Fig. 1 *A* and *B* and *SI Appendix*, Fig. S1 and Table S1). The ~1 Å improvement in resolution over our previously reported structure, determined under the same biochemical conditions (21), is due to microscope hardware and data processing improvements (32, 33). As expected, the structure shows closed extracellular and intracellular gates (Fig. 1*B*). The extracellular C-type gate at the selectivity filter is indistinguishable from our previous structure (*SI Appendix*, Fig. S2). In contrast, we resolved two previously unrecognized features of the intracellular gate in the improved structure: a novel conformation of TM4s that seals the cytoplasmic entrance to the channel and lipids bound inside the channel cavity that directly block the conduction path (Fig. 1*B* and *SI Appendix*, Fig. S3).

Intracellular pH gating involves conformational changes of the inner halves of TM4s including the primary proton sensor K245 (3, 21) (Fig. 1 *B*–*H*). At pH 8.5, TM4 is helical and K245 projects up, away from the cytosol and parallel to the ion conduction axis, toward P124 on TM2 of the opposing subunit (Fig. 1 *B* and *E* and *SI Appendix*, Fig. S4) (21). At pH 6.5, the helical segment between residues 241 to 246 is broken and redirected laterally across the cytoplasmic channel entrance, forming a short intersubunit beta sheet stabilized by a pair of backbone hydrogen bonds between N243 and K245 from opposing subunits (Fig. 1 *C*–*E*). Hydrophilic side chains from N243 and protonated K245 are positioned away from the channel cavity and extend into the cytoplasm, while hydrophobic residues V242, W244, and V246 are positioned on the upper face of the sheet to create a hydrophobic “floor” inside the channel cavity (Fig. 1 *E* and *G*). The cryo-EM map is less well defined following S247, suggesting increased conformational flexibility, but appears in unsharpened maps to resume helical character in a domain-swapped position on the opposite side of the channel relative to open TASK-2_WT_ and all other K2P structures (Fig. 1 *C*–*E*). The interaction between residues 241 to 246 from each subunit seals the cavity from the cytoplasm like a zipper. It is functionally analogous to, but mechanistically and structurally distinct from, the intracellular “X” gate in TASK-1 (34–37) and “Y” gate in THIK-1 channels (38–41) (*SI Appendix*, Fig. S4). We refer to this as a “Z” (for zipper) gate.

The improved low pH structure also shows strong density consistent with lipids protruding into the channel cavity from the membrane inner leaflet (Fig. 1 *A*, *B*, *F*, and *G*). On each side of the channel, an “upper” cavity lipid extends acyl chains through a fenestration between TM2 and TM4 to beneath the selectivity filter and a “lower” cavity lipid acyl chain packs between the upper cavity lipid and the hydrophobic floor of the Z-gate (Fig. 1 *A*, *B*, and *G*). The presence of these acyl chains in the cavity directly occludes the conduction pathway (Fig. 1*F*). The well-defined upper cavity lipids interact with a hydrophobic scaffold composed of L120, V123, L127, L201, L235, L238, and F241 that lines the fenestration entrance and channel cavity (Fig. 1*G*). The lower cavity lipid packs in a hydrophobic groove formed by residues V242, W244, V246 on the Z gate, L127 on TM2, and the upper cavity lipid acyl chain (Fig. 1*G*). Comparing open and closed structures suggests channel opening disfavors lipid binding in two ways. First, it removes the hydrophobic floor for lipid interaction with the top of the zipper gate due to rotation of W244 and V246 laterally toward TM3 (Fig. 1 *E* and *G*). Second, rotation of K245 up toward P124 upon deprotonation partially seals the membrane-facing fenestration and would sterically clash with the lower lipid acyl chain (Fig. 1 *G* and *H*). The fenestration also becomes too narrow for acyl chain access in the open state (minimum radius < 2.0 Å, cross-sectional area < 12 Å^2^, calculated with HOLE using van der Waals radii)(21), though the hydrophobic scaffolding that supports the upper cavity lipid is nearly unchanged (Fig. 1*G*). Subtle rearrangements from the open state to widen the fenestration could thus lead to lipid binding and pore block without requiring K245 protonation and Z-gate formation.

### Introduction of a Polar Residue to the Channel Cavity Results in a Loss of Lipid-Gating.

**Fig. 2.**
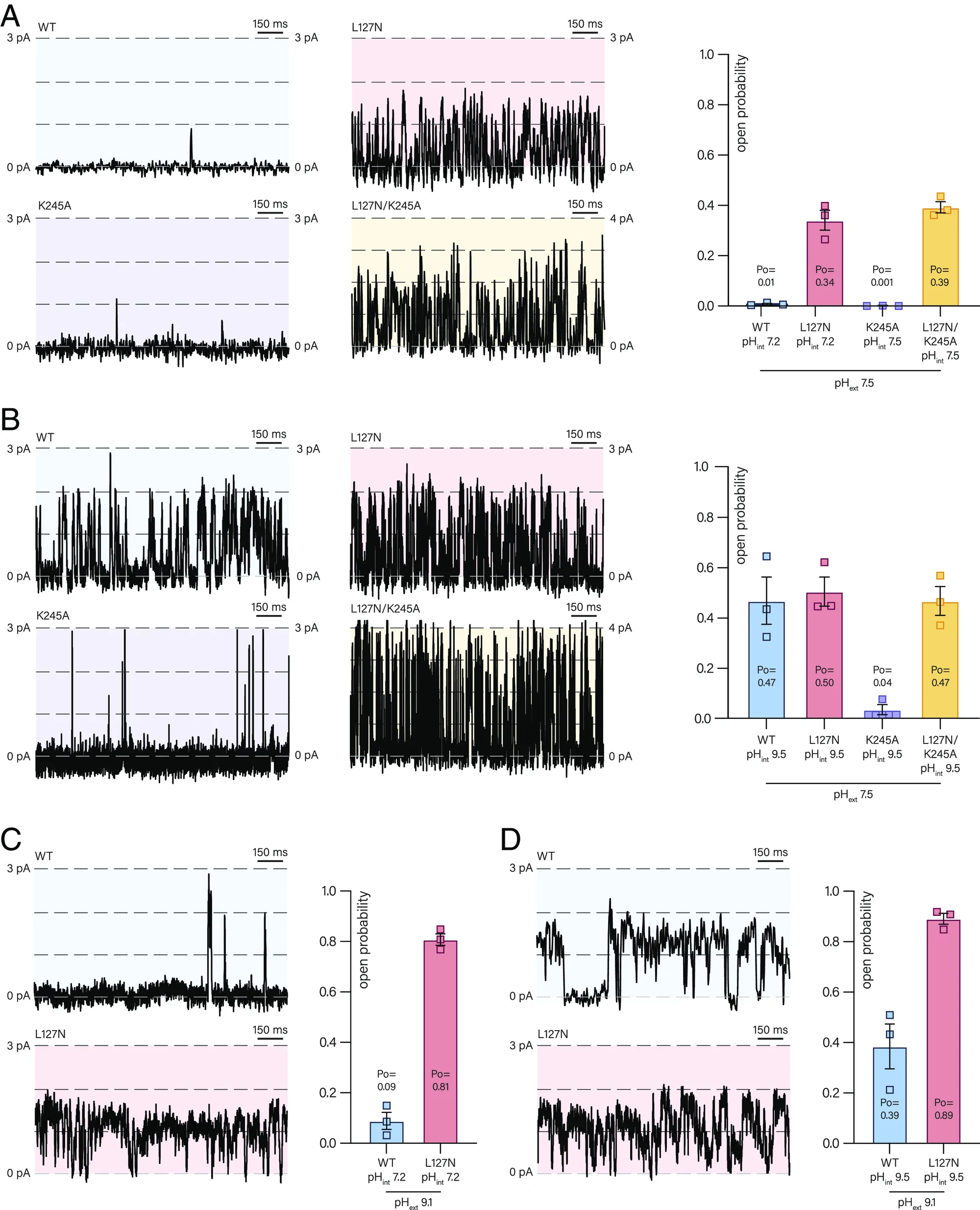
Functional analysis of wild-type (WT) and mutant TASK-2 channels. (*A*) Representative single-channel recordings (*Left*) and mean open probability (*Right*) of TASK-2 constructs at neutral pH_int_ and pH_ext_ (P_o_ = 0.01 ± 0.003, 0.34 ± 0.04, 0.001 ± 0.0002, 0.39 ± 0.02, for TASK-2_WT_, TASK-2_L127N_, TASK-2_K245A_, and TASK-2_L127N/K245A_, respectively, mean ± SEM, n = 3). (*B*) Representative single-channel recordings (*Left*) and mean open probability (*Right*) of TASK-2 constructs at basic pH_int_ and neutral pH_ext_ (P_o_ = 0.47 ± 0.09, 0.50 ± 0.06, 0.03 ± 0.02, 0.47 ± 0.06, for TASK-2_WT_, TASK-2_L127N_, TASK-2_K245A_, and TASK-2_L127N/K245A_, respectively, n =3). (*C*) Representative single-channel recordings (*Left*) and mean open probability (*Right*) of TASK-2_WT_ and TASK-2_L127N_ at neutral pH_int_ and basic pH_ext_ (P_o_ = 0.09 ± 0.03, 0.81 ± 0.02 for TASK-2_WT_ and TASK-2_L127N_ respectively, n = 3). (*D*) Representative single-channel recordings (*Left*) and mean open probability (*Right*) of TASK-2_WT_ and TASK-2_L127N_ at basic pH_int_ and pH_ext_. (P_o_ = 0.39 ± 0.09, 0.89 ± 0.02 for TASK-2_WT_ and TASK-2_L127N_ respectively, n = 3). All recordings were performed at a holding potential of 0 mV in a 10-fold [K^+^] gradient.

We assessed the structural and functional properties of channel mutants to understand the contributions of lipid block and the Z-gate to channel gating. In the closed TASK-2_WT_ structure, L127 directly contacts cavity lipid acyl chains (Fig. 1*G*). TASK-2_L127N_ was identified as a gain-of-function mutation that increased macroscopic channel currents and reduced activation by alkaline pH_int_ (42). We reasoned that introduction of polar asparagines on both sides of the cavity would disfavor lipid binding and prevent occlusion of the conduction pathway. To test this, we first performed single channel recordings. These showed gain of function in TASK-2_L127N_ is predominantly due to increased open probability. TASK-2_L127N_ has a ~40x higher P_O_ than TASK-2_WT_ at neutral pH (TASK-2_L127N_ P_o_ = 0.34 ± 0.04, TASK-2_WT_ P_o_ = 0.01 ± 0.003 at pH_int_ 7.2/pH_ext_ 7.5) (Fig. 2*A*). TASK-2_WT_ P_O_ is increased ~10-fold by external alkalinization (P_o_ = 0.09 ± 0.03 at pH_int_ 7.2, pH_ext_ 9.1) and ~50-fold by internal alkalinization (P_o_ = 0.47 ± 0.09 pH_int_ 9.5/pH_ext_ 7.5) (Fig. 2 *B* and *C*). Alkalinization of both solutions (pH_int_ 9.5/pH_ext_ 9.1) does not further increase open probability above that achieved by internal alkalinization alone (Fig. 2*D*). In contrast, TASK-2_L127N_ P_O_ is increased just ~2.5 fold by external alkalinization (0.81 ± 0.02 at pH_int_ 7.2/pH_ext_ 9.1), ~1.5-fold by internal alkalinization (0.50 ± 0.06 at pH_int_ 9.5/pH_ext_ 7.5), and ~2.6-fold by alkalinization of both solutions (0.89 ± 0.02 at pH_int_ 9.5/pH_ext_ 9.1) (Fig. 2 *B*–*D*). While TASK-2_WT_ openings were not detected under acidic conditions, TASK-2_L127N_ showed clear activity indicative of reduced inhibition by extracellular and intracellular protons, with a more pronounced defect in intracellular gating: P_O_ was 0.76 ± 0.11 at pH_int_ 6.2/pH_ext_ 9.1, 0.24 ± 0.01 at pH_int_ 9.5/pH_ext_ 6.5, and 0.03 ± 0.02 at pH_int_ 6.5/pH_ext_ 6.5 (*SI Appendix*, Fig. S5). Notably, TASK-2_WT_ P_O_ is limited under maximally activating conditions to ~0.5 by long duration closures (t ≥ 100 ms) that interrupt bursts of channel opening (Fig. 2*D* and *SI Appendix*, Fig. S5). These long duration closures are absent in TASK-2_L127N_ such that P_O_ approaches 1 under the same conditions (Fig. 2*D* and *SI Appendix*, Fig. S5).

**Fig. 3.**
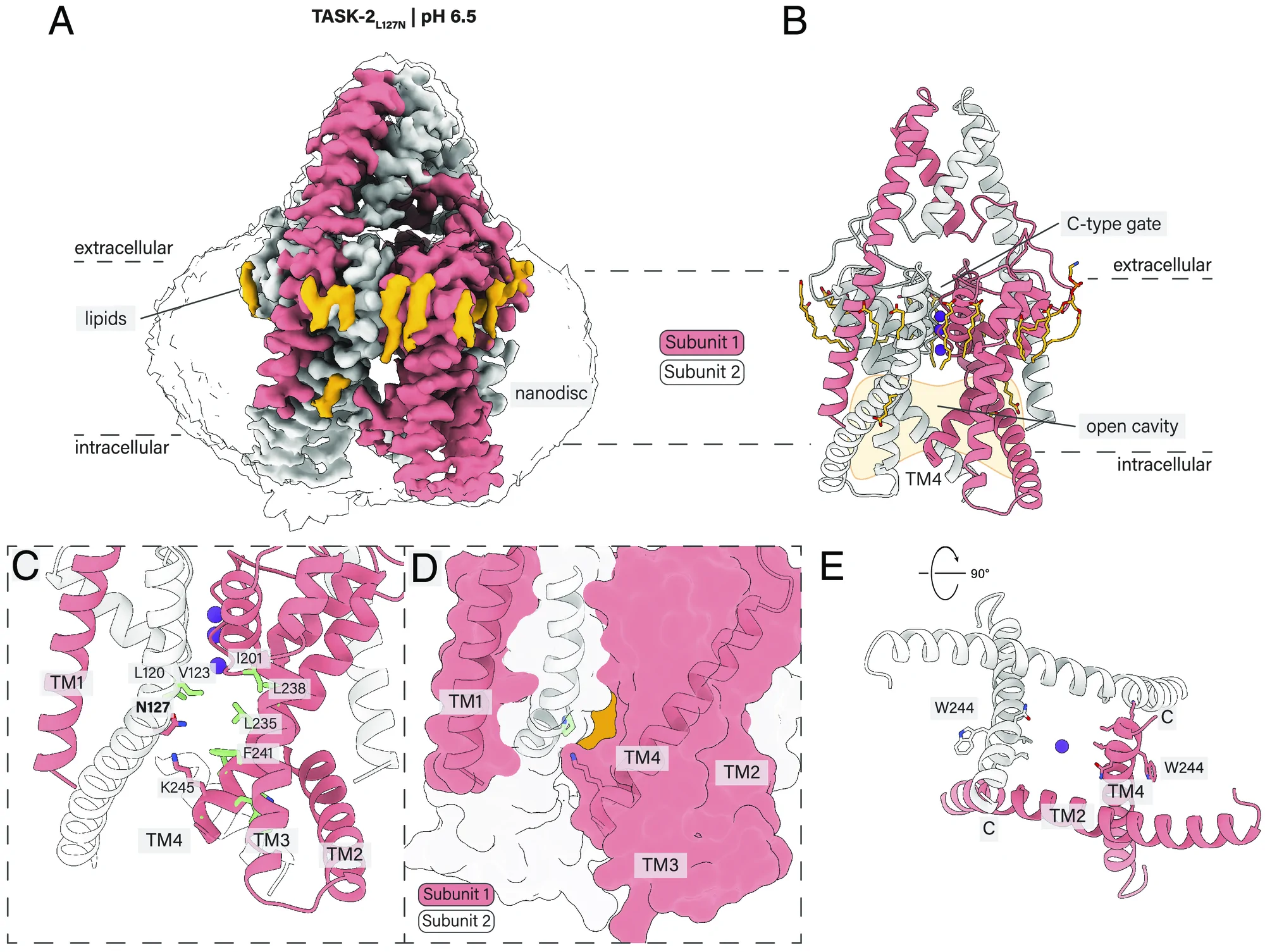
Structural basis for gain of function in TASK-2_L127N_. (*A*) Side view of TASK-2_L127N_ pH 6.5 cryo-EM map and (*B*) pH 6.5 closed channel model. Channel subunits are red and white, K^+^ is purple, and lipids are gold. Side views of (*C*) model and (*D*) protein surface highlighting lateral fenestration and cavity environment. (*E*) Cytosolic view of TASK-2_L127N_ at pH 6.5 with open Z-gate and cavity.

We determined a cryo-EM structure of TASK-2_L127N_ in lipid nanodiscs at pH 6.5 to an overall resolution of 2.8 Å to investigate the consequences of the mutation on lipid block and Z-gating (Fig. 3 *A* and *B* and *SI Appendix*, Fig. S6 and Table S1). The TASK-2_L127N_ extracellular gate is closed, just as observed in the closed TASK-2_WT_ structure (*SI Appendix*, Fig. S2). In contrast, the intracellular side of the channel adopts an open conformation similar to TASK-2_WT_ at high pH: Lipid density is absent from the channel cavity, K245 residues project up from the top of TM4 away from the cytosol and parallel to the ion conduction axis, and the Z-gate is open (Fig. 3 *C* and *D* and *SI Appendix*, Fig. S4). The only notable difference in the intracellular sides of TASK-2_L127N_ at pH 6.5 and TASK-2_WT_ at pH 8.5 is a lack of defined density after V246 in TASK-2_L127N_, presumably due to increased flexibility in the C-terminal ends of TM4 (*SI Appendix*, Fig. S6). We conclude that gain of function in TASK-2_L127N_ is a result of disrupted internal gating. N127 disfavors lipid binding in the channel cavity through direct electrostatic repulsion and indirectly disfavors Z-gate closure, probably through loss of stabilizing cavity lipids. Loss of long closures in TASK-2_L127N_ under maximally activating conditions that limit TASK-2_WT_ open probability suggests they are a consequence of lipid block and/or Z-gate closure even at high pH_int_.

### Lipid Block Does Not Require Z-Gate Closure.

We next characterized a mutation of the primary internal proton sensor, TASK-2_K245A_. This mutation is expected to compromise Z-gating if gate closure is promoted by unfavorable electrostatic interactions between protonated K245 ε-amino groups and the hydrophobic environment around TM2 and inner leaflet lipids, but it is not expected to restrict lipid access to the cavity. Macroscopic recordings show reduced sensitivity to internal proton inhibition (*SI Appendix*, Fig. S7 *A*–*C* and Table S3). These experiments were designed to measure pH dose–response relationships, which are normalized within each patch and are independent of channel number. Maximal current amplitudes are not comparable between constructs, as macroscopic current reports the product of channel number, open probability, and unitary current, and channel number was neither controlled nor quantified (*Methods Summary*). We therefore measured open probability directly (Fig. 2 and *SI Appendix*, Table S2). In single channel recordings, the most pronounced effect of the mutation is a much (~10 to 15-fold) lower open probability than TASK-2_WT_ in both neutral (P_o_ = 0.001 ± 0.0002 at pH_int_ 7.5/pH_ext_ 7.5) and alkaline internal conditions (P_o_ = 0.03 ± 0.02 at pH_int_ 9.5/pH_ext_ 7.5) (Fig. 2 *A* and *B*).

**Fig. 4.**
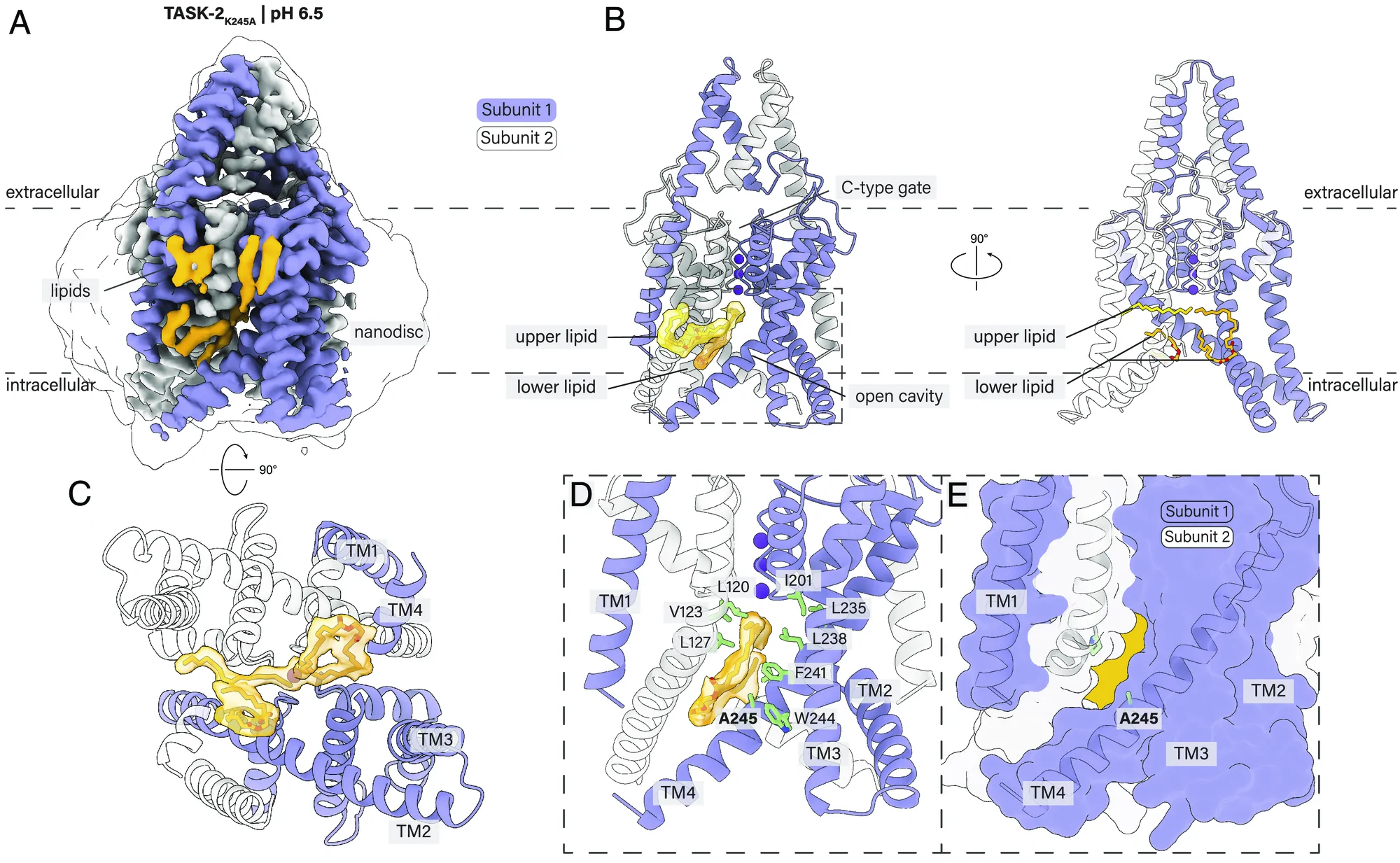
Lipid block of TASK-2 does not require Z-gate formation. (*A*) Side view of TASK-2_K245A_ pH 6.5 cryo-EM map and (*B*) pH 6.5 occluded channel model with cavity lipids and carved cryo-EM density (*Left*) and rotated view of cavity lipids (*Right*). Channel subunits are purple and white, K^+^ is purple, and lipids are gold. (*C*) Cytosolic view of TASK-2_K245A_ at pH 6.5 with cavity lipids and carved cryo-EM density. Side views of (*D*) model and (*E*) protein surface highlighting cavity environment and lateral fenestration (orange).

We determined a cryo-EM structure of TASK-2_K245A_ reconstituted in lipid nanodiscs at pH 6.5 to an overall resolution 3.2 Å (Fig. 4*A* and *SI Appendix*, Fig. S8 and Table S1). The structure shows a closed extracellular gate (*SI Appendix*, Fig. S2) and open intracellular Z-gate (Fig. 4 *B* and *C* and *SI Appendix*, Fig. S4). In contrast to other structures with open Z-gates (TASK-2_WT_ at high pH and TASK-2_L127N_ at low pH), we observe clear density for lipids in the channel cavity that block the TASK-2_K245A_ conduction path (Fig. 4 *B*–*D*). Lipids are present on each side of the channel with acyl chains projecting into the cavity below the selectivity filter to interact with the same residues that form the hydrophobic scaffold in closed TASK-2_WT_ (Figs. 1*F* and 4*D*). Truncation of the K245 side chain in TASK-2_K245A_ expands membrane-facing gaps between TM2s and TM4s, which are partially sealed by the lysine aliphatic chain in TASK-2_WT_ (Fig. 4*E*). This expansion is likely to promote lipid access without TM4 rearrangement and Z-gate formation, explaining the reduced open probability of TASK-2_K245A_ in neutral and alkaline pH_int_ conditions. These results show that zipper gate formation is not strictly required for cavity lipid binding and block. This suggests that by acting as a partial membrane seal at high pH, K245 disfavors lipid access to the channel interior, thereby promoting activity, an idea supported by prior molecular dynamics simulations (30).

Our model predicts that L127N, which directly disfavors lipid block, and K245A, which directly disfavors Z-gate formation, would have a synergistic effect and further compromise internal gating. Indeed, single-channel recordings of TASK-2_L127N/K245A_ show no appreciable cytosolic proton-mediated gating (Fig. 2 *A* and *B* and *SI Appendix*, Fig. S5 and Table S2). Varying internal pH from 6.5 to 9.5 in constant neutral external pH did not significantly change open probability (P_o_ = 0.43 ± 0.03, 0.39 ± 0.02, 0.47 ± 0.06, for pH_int_ 6.5/pH_ext_ 7.5, pH_int_ 7.5/pH_ext_ 7.5, and pH_int_ 9.5/pH_ext_ 7.5, respectively) (Fig. 2 *A* and *B* and *SI Appendix*, Fig. S5). While open probability is insensitive to pH_int_, mean single channel conductance is ~twofold lower at acidic pH_int_ compared to basic pH_int_ (Fig. 2 *A* and *B* and *SI Appendix*, Fig. S12), which explains the ~twofold increase in macroscopic currents recorded within individual excised patches in response to pH_int_ changes (*SI Appendix*, Fig. S7 *A*–*C* and Table S3). The double mutant shows reduced efficacy of pH_ext_ gating, with incomplete inhibition by acidic external conditions (~25% I/I_max_ at pH_ext_ 5.5), indicative of potential allostery between inner and outer gate closure (*SI Appendix*, Fig. S7). That pH_int_ gating is lost in TASK-2_L127N/K245A_, but not in TASK-2_L127N_ or TASK-2_K245A_ alone, suggests Z-gate formation and lipid block are both pH-dependent processes (Fig. 2). Residual inner gating in TASK-2_K245A_ is likely due to a pH dependence of cavity lipid binding, while in TASK-2_L127N_ it is likely due to pH-dependent Z-gate closure.

### A Local Anesthetic Binds in the Channel Cavity to Inhibit TASK-2.

**Fig. 5.**
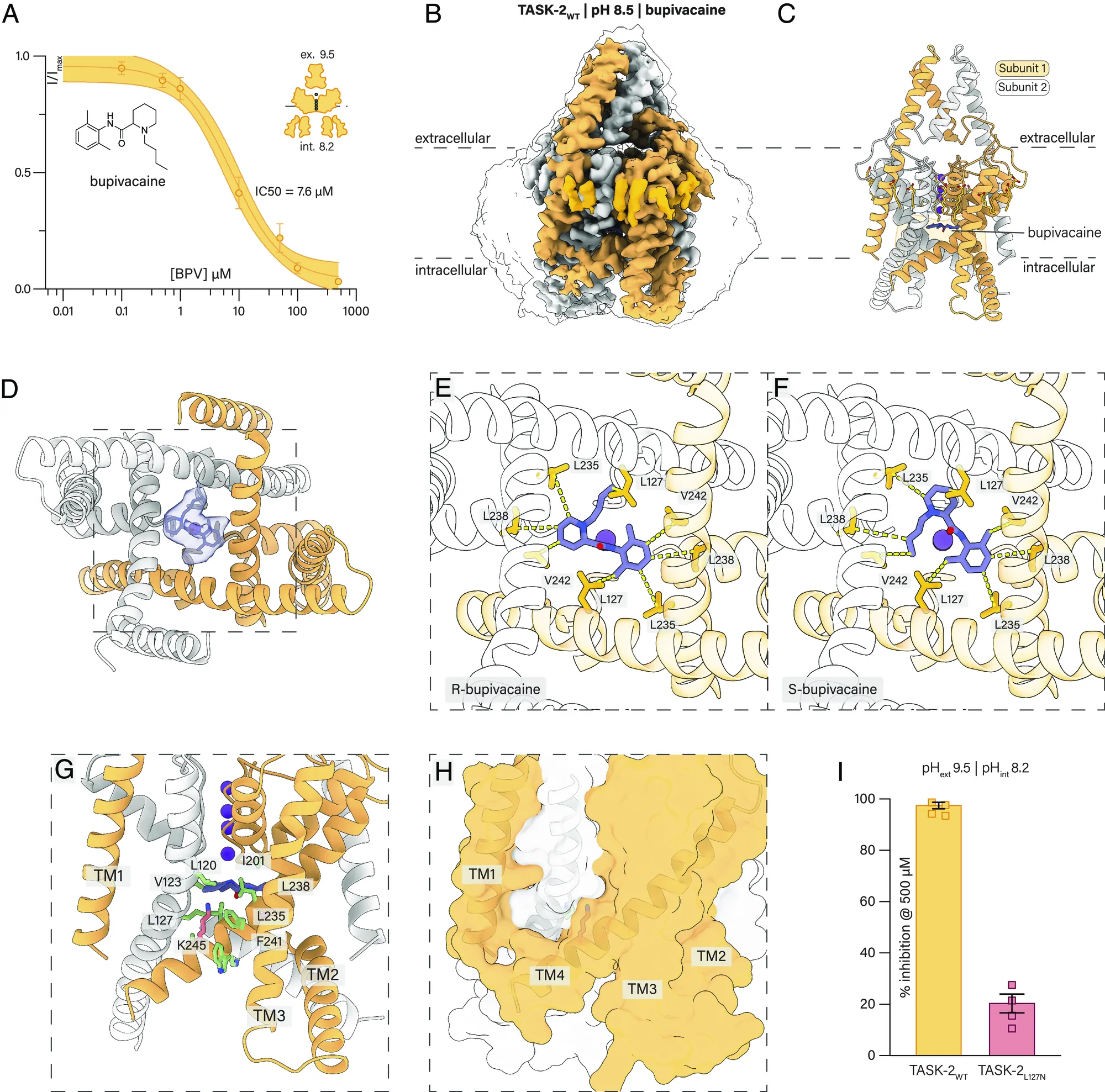
Structural basis for bupivacaine inhibition of TASK-2. (*A*) Dose response from excised patches of TASK-2_WT_ treated with increasing concentrations of bupivacaine at pH_int_ 8.2 and pH_ext_ 9.5. Fit to Boltzmann relationship is shown with 95% CI (IC_50_ = 7.6 ± 0.91 µM, mean ± SEM, n = 4 patches). (*B*) Side view of TASK-2_WT_ pH 8.5 with 300 µM bupivacaine cryo-EM map and (*C*) bupivacaine occluded channel model. Channel subunits are orange and white, K^+^ is purple, lipids are gold, and bupivacaine is shown in purple. (*D*) Cytosolic view with bupivacaine and carved cryo-EM density. One modeled bupivacaine pose is shown. (*E* and *F*) One R-bupivacaine (*E*) and S-bupivacaine (*F*) binding pose with associated hydrophobic interactions. Side views of (*G*) model and (*H*) protein surface highlighting cavity environment and lateral fenestration (orange). (*I*) Comparison of TASK-2_WT_ and TASK-2_L127N_ inhibition by bupivacaine recorded at 0 mV. Recordings in (*A* and *I*) were performed at a holding potential of 0 mV in a 10-fold [K^+^] gradient.

Molecular mechanisms for pharmacological inhibition of TASK-2 are unknown. The local anesthetic bupivacaine is among the highest affinity TASK-2 inhibitors reported (28), with similar apparent affinity for TASK-2 and voltage-gated sodium channel targets (43). Racemic bupivacaine inhibited mouse TASK-2 with an IC_50_ = 7.6 ± 0.91 µM (n = 4 patches) under conditions where both inner and outer gates are predominantly open (pH_int_ 8.2/pH_ext_ 9.5, Fig. 5*A*). We determined the structure of TASK-2_WT_ in lipid nanodiscs at pH 8.5 in the presence of bupivacaine to an overall resolution of 3.0 Å (Fig. 5*B* and *SI Appendix*, Fig. S9). The structure shows open inner and outer pH gates like the open TASK-2_WT_ structure determined under the same conditions (Fig. 5*C* and *SI Appendix*, Figs. S2 and S4). An additional strong, nonprotein, density feature in the channel cavity underneath the selectivity filter is consistent with bupivacaine bound in four possible configurations, each with 0.25 occupancy (R- or S-bupivacaine in two poses each related by a ~180° rotation); each channel can sterically accommodate the binding of one molecule of bupivacaine (Fig. 5 *C*–*H*). In all poses, bupivacaine bridges opposing TM2s and TM4s through interactions with V123, C126, L127, I130, L235, L238, S239, and V242. These interactions pull TM4 up toward the extracellular solution, constricting the lateral fenestration relative to TASK-2_WT_ at high pH (Fig. 5 *G* and *H*). While distinguishing density for weakly coordinated ions at these resolutions is challenging, we note that bupivacaine-bound TASK-2 at pH 8.5 lacks the density observed for the external K+ coordination site S0, which sits above the selectivity filter, and is modeled with ions at S1 to S4, whereas apo TASK-2 at pH 8.5 is modeled with ions at S0 to S4. This is despite the two channels adopting essentially the same conformation, within the error of the coordinates, in the surrounding selectivity filter and helical cap (rmsd = 0.6 Å, *SI Appendix*, Fig. S2). For comparison, the closed structures determined at pH 6.5 all show density supporting ions at S2, S3, and S4 but not at S1 or S0, consistent with disruption of the outermost coordination sites upon closure of the C-type external gate (*SI Appendix*, Fig. S2).

This structural model for bupivacaine inhibition by cavity binding and pore block predicts TASK-2_L127N_ would show reduced inhibition due to unfavorable interactions between polar asparagine side chains in the cavity and apolar aspects of the drug. Consistently, we found TASK-2_L127N_ is nearly insensitive to bupivacaine block, with only modestly reduced currents at high drug concentrations [16.7 ± 2.0% inhibition at 500 µM, (n = 3)] that completely inhibit TASK-2_WT_ (Fig. 5*I*). Since the binding site for bupivacaine overlaps with that for cavity lipids, we reasoned bupivacaine binding would be reduced at low pH_int_ when lipid occupancy is high. To test this, we added 300 µM bupivacaine to a sample of TASK-2_WT_ in lipid nanodiscs at pH 6.5 and determined a cryo-EM structure of the channel to an overall resolution of 2.9 Å. Indeed, the structure is nearly identical to the apo closed TASK-2_WT_ structure determined under the same conditions, with a closed outer gate, closed Z-gate, and lipids bound in the channel cavity, with no evidence for bupivacaine binding (*SI Appendix*, Figs. S10 and S11).

## Discussion

**Fig. 6.**
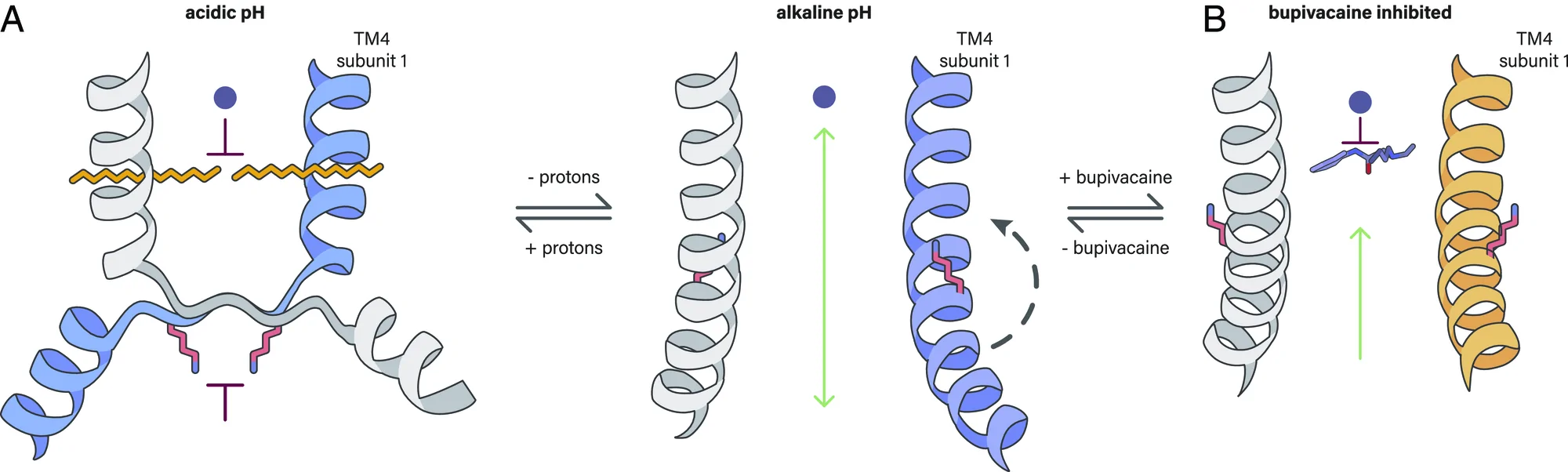
Model for TASK-2 intracellular gating and inhibition. (*A*) Model for TASK-2 pH_int_ gating through Z-gate formation and lipid block. Z-gate formation creates. a hydrophobic floor within the inner channel cavity and opens lateral fenestrations for lipid entry and channel block. Deprotonation of K245 opens the Z-gate and increases TM4 helicity such that K245s partially occlude lateral fenestrations to disfavor lipid binding. (*B*) Model for channel inhibition by bupivacaine. Bupivacaine binds the open channel cavity at a site that overlaps with lipids at low pH_int_ to block ion conduction.

Our data support a model for TASK-2 channel gating and inhibition illustrated in Fig. 6. Extracellular pH regulation of the channel operates through a C-type gate as previously reported (21) with protonation of R224 at the top of TM4 causing conformational changes that disrupt selectivity filter structure and ion coordination at sites S0 and S1 (*SI Appendix*, Fig. S2). Intracellular pH gating involves stacked lipid- and Z-gates. At high pH_int_, K245 projects up from TM4 toward the hydrophobic bottom face of TM2 and partially seals the lateral fenestrations between TM4 and TM2 that separate the channel cavity from the membrane. When K245 is protonated, a portion of each TM4 unwinds, projects across the channel, and forms an intersubunit beta sheet that seals the mouth of the cavity from the cytoplasmic solution like a zipper. Movement of K245 during Z-gate formation opens the lateral fenestrations to permit high occupancy binding of acyl chains from inner leaflet lipids inside the hydrophobic cavity between the top of the Z-gate and bottom of the selectivity filter. Mutational analysis shows both aspects of the intracellular gate contribute to pH_int_ regulation. TASK-2_L127N_ results in gain of function by directly disfavoring lipid block, eliminating long-duration closures that impose a ceiling on WT open probability of ~0.5, and indirectly destabilizing Z-gate formation. TASK-2_K245A_ prevents Z-gate formation, but reduces open probability by creating larger lateral fenestrations that promote lipid access to the channel cavity and pore block. Both Z-gate formation and lipid block are likely to be intrinsically pH-sensitive as a loss of intracellular proton inhibition is only observed in the double mutant TASK-2_L127N/K245A_.

A previous study (3) reported higher activity for TASK-2_K245A_ in excised patches than shown here (Fig. 2 and *SI Appendix*, Figs. S7 and S12 and Tables S2 and S3). This is likely explained by differences in recording conditions. Those recordings were made at −60 mV in high K^+^_ext_ (140 mM), conditions that promote an open external pH gate, while ours were made at 0 mV in lower K^+^_ext_ (15 mM). Whole cell recordings of TASK-2_K245A_ from both groups under the same conditions showed similar pH_int_ sensitivity (3, 21). More broadly, we note macroscopic current magnitudes do not always track single channel open probability across constructs studied here and previously (Fig. 2 and *SI Appendix*, Fig. S7 and Tables S2–S4) (3, 21). We did not assess or control for expression levels in macroscopic recordings, and differences in channel number, trafficking, or regulatory interactions between constructs may contribute to this apparent discrepancy (*SI *Materials and* Methods*). Absolute macroscopic current amplitudes are therefore uninformative about open probability, and the functional conclusions drawn here rely on single channel open probabilities and on pH dose responses normalized within individual patches or cells.

Pore block by cavity bound lipids through lateral membrane openings or fenestrations may be a common aspect in K2P channel gating. Lipid or detergent acyl chains are present in closed TRAAK structures and mutational studies support a model in which lipid binding and unbinding set leak activity, while movement of TM4 upon mechanical stimulation seals lateral openings and prevents block (23, 24). Inhibitory lipids were similarly observed inside the cavity of TREK-1 (25). Structural studies also identified lipid or detergent acyl chains in the cavities of TWIK-1 (22, 26) and TWIK-2 (27) that are suggested to contribute to gating. Cavity lipids in K2Ps are expected to similarly plug the pore to block conduction, though their precise binding mode may differ among channels. TRAAK, TWIK-1, and TASK-2 are modeled similarly with inner leaflet lipid acyl chains accessing the cavity through fenestrations to bind under the filter; in contrast, the lipid headgroups are modeled in this position in TREK-1 and TWIK-2. Notably, introduction of polar mutations into the cavity of many K2Ps at the same position of TASK-2_L127N_ (position TM2.6) increases channel activity, including in all vertebrate channels, *Drosophila* KCNK0/ORK1, and C. elegans TWK-18 (42). In previous work, we hypothesized an analogous mutation in TRAAK (TRAAK_G158D_) causes gain-of-function by disfavoring lipid block of the cavity (24) based on structural data, showing TRAAK_G158D_ lacked cavity-bound pore-blocking acyl chains present in WT TRAAK, and single channel recordings, showing TRAAK_G158D_ lacks long closures and promotes leak-type openings. Based on these results and data presented here, we speculate mutations at this site could similarly disrupt lipid block to activate other K2Ps.

Still, the link between cavity lipid occupancy in structures and channel activity is correlative, and we cannot exclude alternative mechanisms to explain TASK-2_L127N_ gain-of-function. In addition to disrupted lipid block, TM2.6 mutant gain-of-function has been proposed to result from allosteric stabilization of a selectivity filter gate, pore-dewetting and disruption of a hydrophobic vapor barrier, or other changes not captured in static structures (42). These models are not necessarily mutually exclusive. A hydrophobic barrier underlying pore closure may correspond to a dewetted empty cavity, a cavity occluded by a bound lipid acyl chain, or a continuum between these states; in all cases, polar substitutions at TM2.6 would promote cavity hydration and could in turn stabilize a conductive selectivity filter. We further cannot exclude the possibility that acyl chain density in K2P structures reflects, at least in part, an artifact of membrane extraction during purification. Still, we favor disrupted lipid block as the most parsimonious explanation for current data, including the observation of cavity acyl chains in multiple K2P structures from different groups in different detergents and lipid nanodiscs, the loss of acyl chain density in TRAAK and TASK-2 with TM2.6 polar mutations, and the electrophysiological signatures of these of mutants in single channel recordings (22–27).

Cavity binding and pore block is similarly emerging as a common mechanism for pharmacological inhibition of K2Ps. Cavity binding sites for inhibitors have been characterized in three K2P subfamilies: cannabidiol in TRAAK and TREK-1 channels (44), pimozide in TWIK-2 (45), and BAY1000493/2341237 in TASK-1 (36). We found that bupivacaine inhibits TASK-2 by binding in the channel cavity at a site that overlaps with that of cavity lipids at low pH_int_. Bupivacaine binding is only observed at high pH_int_, consistent with state-dependent block that requires an open intracellular gate. This is likely explained by competition for the site by bound lipids and reduced access of the drug to the cavity at low pH. Bupivacaine is a weak base (pKa = 8.1). At pH 8.5, when the majority of the drug is uncharged, bupivacaine can access the cavity through the membrane; at low pH, when bupivacaine is predominantly charged, membrane access is less favorable and the closed TASK-2 Z-gate prevents access to the cavity from the cytoplasm. The bupivacaine binding site in TASK-2 is notably different from that experimentally determined in Na_v_1.7 and predicted for TASK-1. In Na_v_1.7, bupivacaine binds between S6 helices below the intracellular gate at the cytoplasmic entrance to the channel (29). In TASK-1, bupivacaine is predicted to bind inside membrane facing fenestrations, akin to fluoxetine binding sites in TREK-2 (31, 46).

Our results provide insight into the strikingly different gating mechanisms of K2P channels. TASK-2, TWIK-1, and TASK-1/-3 are all inhibited by extracellular protons through C-type gates, but they utilize completely distinct protons sensors and selectivity filter rearrangements that result in different ranges in pH sensitivity (22, 34, 35, 37). TASK-1, THIK-1, and TASK-2 all utilize intracellular protein constrictions to gate closed, but their X-, Y-, and Z-gates are structurally unique and only TASK-2 has been shown to additionally involve lipid block (34–41). We expect the unique aspects of TASK-2 intracellular gating and inhibition revealed here could facilitate discovery and development of specific, high affinity pharmacological agents for the treatment of TASK-2 related disorders.

## Methods Summary

*Mus musculus* TASK-2 (amino acids 1 to 335, with or without mutations) was expressed in *Pichia pastoris*, extracted in DDM/CHS, purified by anti-GFP nanobody affinity chromatography and size-exclusion chromatography, and reconstituted into MSP1D1 nanodiscs with a 2:1:1 DOPE:POPS:POPC lipid mixture for structural studies at pH 8.5 or pH 6.5, with or without 300 µM bupivacaine. Cryo-EM data were collected on a Titan Krios or Talos Arctica and processed in RELION and cryoSPARC with iterative TOPAZ particle picking, 2D/3D classification, Bayesian polishing, and nonuniform refinement to yield final reconstructions at 2.6 to 3.2 Å resolution (47–50). Atomic models were built by rigid-body fitting prior TASK-2 structures (PDB 6WLV, 6WM0) followed by iterative manual rebuilding in Coot, real-space refinement in Phenix, and validation with Molprobity (51–54). Electrophysiological characterization was performed on *M. musculus* TASK-2 expressed in *Xenopus laevis* oocytes using inside-out patch-clamp (single-channel and macroscopic) or two-electrode voltage-clamp recordings (55). Data are reported as mean ± SEM and compared by one-way ANOVA with Dunnett’s correction or unpaired *t* tests; full details are provided in *SI Appendix*.

## Supplementary Material

Appendix 01 (PDF)

## Data Availability

Single channel analysis code written in Python for this paper is available on Github: https://github.com/BrohawnLab/ElectrophysiologyScripts/tree/singles (56). Protein structures are deposited in the PDB [accession codes 10OY (57), 10PB (58), 10PF (59), 10PC (60), and 10PD (61] and cryo-EM maps are deposited in the EMDB [accession codes 75365 (62), 75367 (63), 75371 (64), 75368 (65), and 75369 (66)].
