# Supplementary material for: Mechanisms of intracellular pH gating and local anesthetic inhibition of the two-pore domain K+ channel TASK-2
Source: Proc Natl Acad Sci U S A. 2026 Oct 8;123(41):e2604873123. doi: 10.1073/pnas.2604873123 (PMC13663809; doi:10.1073/pnas.2604873123)
Supplement: Supplementary file 1 [file pnas.2604873123.sapp.pdf]

Supporting Information

Mechanisms of intracellular pH gating and local anesthetic inhibition of the two-pore domain K^+ channel TASK-2

Trevor Docter[#1-3], Ben Sorum[#†1-3], Baobin Li[#1-3,‡], Robert A. Rietmeijer[1-3,5], Annan S.I. Cook[1,5], Abhay Kotecha[6,§], and Stephen G. Brohawn[1-5]

[1]Department of Molecular & Cell Biology, University of California Berkeley, Berkeley, California 94720, USA.

[2]Department of Neuroscience, University of California Berkeley, Berkeley, California 94720, USA.

[3]Helen Wills Neuroscience Institute, University of California Berkeley, Berkeley, California 94720, USA.

[4]California Institute for Quantitative Biology (QB3), University of California, Berkeley, CA 94720, USA.

[5]Biophysics Graduate Program, University of California, Berkeley, CA 94720, USA.

[6]Materials and Structural Analysis, Thermo Fisher Scientific, Eindhoven, the Netherlands.

[†]Current address: Department of Biomedical Sciences, Cooper Medical School of Rowan University, Camden, NJ, 08103, USA.

[‡]Current address: Department of Anesthesiology, Zhongshan Hospital, Institute for Translational Brain Research, State Key Laboratory of Brain Function and Disorders, MOE Frontiers Center for Brain Science, Fudan University, Shanghai, China.

[§]Current address: Department of Structural Biology, St. Jude Children's Research Hospital, Memphis, TN, 38105, USA.

[#]Equal contribution

*Correspondence to: brohawn@berkeley.edu

This PDF file includes:

SI Materials and Methods

Tables S1-4

Figures S1-S12

SI References

SI Materials and Methods

Cloning, expression and purification

Cloning, expression and purification were performed similarly to that previously described for TASK-2[1]. A gene encoding *Mus musculus* TASK-2 (Uniprot: Q9JK62) was codon-optimized for expression in *Pichia pastoris*, synthesized (Genewiz, Inc.) and cloned into a modified pPICZ-B vector (Life Technologies, Inc.). The resulting construct encoded a human rhinovirus 3C protease-cleavable C-terminal eGFP-10x histidine fusion protein. A truncation of the C-terminal 167 amino acids, which are predicted to be largely unstructured, was found to improve protein expression and biochemical stability.

The resulting construct, TASK-2(aa1-335)-SNS-LEVLFQ/GP-(EGFP)-HHHHHHHHHH was used for structural studies and is referred to as TASK-2_{WT} in the text for simplicity. The Pme1 linearized pPICZ-B plasmid was transformed into the *P. pastoris* (Invitrogen) strain SMD1163 by electroporation and transformants were selected on YPDS plates with 0.5 mg/ml zeocin. Cells were not authenticated or tested for mycoplasma contamination. The expression levels of individual transformants were analyzed by fluorescence size-exclusion chromatography (FSEC). Large-scale expression from a suitable transformant was performed in MiniFors 2 3L fermenter. Overnight cultures of cells in YPD + 0.5 mg/ml zeocin were added to minimal media to a starting OD600 of ~1 and grown at 30 °C with glycerol feeding for 48 h at 40% O2 saturation and pH 5.0 maintained with variable stir speed, air/O2 gas mixture, gas flow rate and NH_4OH addition. Cells were then starved to deplete glycerol, the temperature was reduced to 27 °C and protein expression was induced by a gradually increasing methanol feed rate. Expression continued for ~48–60 h. Cells were pelleted, flash-frozen in liquid nitrogen and stored at−80 °C. Cells (30 g) were broken by milling (Retsch model MM301) for five cycles of 3 min at 25 Hz. All subsequent purification steps were carried out at 4 °C. Cell powder was added to 100 ml lysis buffer containing 50 mM Tris pH 8.5 (pH values for Tris buffers correspond to values at 4 °C), 150 mM KCl, 1 mM phenylmethylsulfonyl fluoride, 1 mM EDTA, 10 µl benzonase nuclease (EMD Milipore), 1 mM E64, 1 mg/mL pepstatin A, 10 mg/mL soy trypsin inhibitor, 1 mM benzamidine, 1 mg/mL aprotinin, 1 mg/mL leupeptin. The solution was sonicated and centrifuged at 150,000g for 45 min. The supernatant was discarded and the membrane pellet was transferred into a Dounce homogenizer containing extraction buffer (50 mM Tris pH 8.5, 150 mM KCl, 1 mM phenylmethylsulfonyl fluoride, 1 mM EDTA, 10 µL benzo-nase nuclease, 1 µM AEBSF, 1 mM E64, 1 mg/mL pepstatin A, 10 mg/mL soy trypsin inhibitor, 1 mM benzamidine, 1 mg/mL aprotinin, 1 mg/mL leupeptin, 1% n-dodecyl-β-d-maltopyranoside (DDM; Anatrace) and 0.2% cholesterol hemisuccinate Tris salt (CHS; Anatrace)). Membrane pellets were homogenized in 100 mL buffer and then gently stirred at 4 °C for 2 h. The extraction was centrifuged at 33,000g for 45 min. Sepharose resin (5 mL) coupled to anti-GFP nanobody was added to the supernatant and stirred gently for 2 h at 4 °C. The resin was collected in a column and washed with 50 ml Buffer 1 (20 mM Tris, 150 mM KCl, 1 mM EDTA, 0.025% DDM and 0.005% CHS, pH 8.5), 150 ml Buffer 2 (20 mM Tris, 300 mM KCl, 1 mM EDTA, 0.025% DDM and 0.005% CHS, pH 8.5) and20 ml Buffer 1. Human rhinovirus 3C protease (~0.5 mg) was added into the washed resin in 5 mL Buffer 1 and rocked gently overnight. Cleaved TASK-2 was eluted and concentrated to ~0.4 mL with an Amicon Ultra spin concentrator (50 kDa cut-off, Millipore Sigma). The concentrated protein was subjected to size-exclusion chromatography using

a Superdex 200 Increase 10/300 column (GE Healthcare) run in Buffer 3 (20 mM Tris pH 8.5, 150 mM KCl, 1 mM EDTA, 0.025% DDM and 0.0025% CHS) on a NGC system (Bio-Rad) with ChromLab 6. The peak fractions were collected and spin concentrated for reconstitution.

Nanodisc assembly

Freshly purified TASK-2 was reconstituted into MSP1D1 nanodiscs with a 2:1:1 DOPE:POPS:POPC lipid mixture (mol:mol; Avanti) at a final molar ratio of TASK-2:MSP1D1:lipid of 1:5:250. Lipids in chloroform were mixed, dried under argon, washed with pentane, dried under argon, and dried under vacuum overnight in the dark. Dried lipids were rehydrated in buffer 4 containing 20 mM Tris, 150 mM KCl, 1 mM EDTA, pH 8.5, and clarified by bath sonication. DDM was added to a final concentration of 8 mM. TASK-2 was mixed with lipids and incubated at 4 °C for 30 min before the addition of MSP1D1 protein. After incubation for 10 min at 4 °C, 100 mg of Bio-Beads SM2 (Bio-Rad) (prepared by sequential washing in methanol, water and Buffer 4 and weighed damp following bulk liquid removal) was added and the mixture was rotated at 4°C overnight. The sample was spun down to facilitate the removal of solution from the Bio-Beads and the reconstituted channel was further purified on a Superdex 200 increase column run in 20 mM Tris pH 8.5,150 mM KCl and 1 mM EDTA (high pH) or 20 mM potassium phosphate pH 6.5, 150 mM KCl and 1 mM EDTA (low pH). The peak fractions were collected and spin concentrated (50 kDa molecular weight cut-off) to 1.0–1.2 mg/ml for grid preparation. For relevant preparations, 100 mM stock bupivacaine in ethanol was diluted to 3 mM in H_20 then spiked into the nanodisc sample at a 1:10 ratio, granting a final concentration of 300 µM.

Grid preparation and cryo-EM data collection

The TASK-2 nanodisc samples were centrifuged at 21,000g for 10 min at 4 °C immediately prior to grid preparation. 3 µl samples were applied to holey carbon, 300 mesh R1.2/1.3 gold grids (Quantifoil) that were freshly glow discharged for 30 s. Samples were incubated for 5 s at 4 °C and 100% humidity before blotting with Whatman #1 filter paper for 3–3.5 s at blot force 1 and plunge-freezing in liquid ethane cooled by liquid nitrogen using a FEI Vitrobot Mark IV (FEI/Thermo Scientific). Grids were clipped and transferred to a FEI Talos Arctica electron microscope operated at 200 kV (TASK-2_{K245A}) or Titan Krios G4 microscope equipped with a cold field emission gun (CFEG) operated at 300 kV at the Thermo Fisher Scientific RnD division in Eindhoven, Netherlands.

For TASK-2_{K245A}, images were recorded in an automated fashion with SerialEM using image shift with a target defocus range of 0.7-2.2 µm over 5 s as 50 subframes on a K3 direct electron detector (Gatan, USA) with a pixel size of 1.115 Å and a total dose of 50 e− /Å^2. All other datasets were collected on a Falcon 4 detector that was mounted behind a Selectris X energy filter with slit width set to 10 eV. For TASK-2_{WT}, images were collected in super-resolution with a pixel size of 0.3635 Å/px, total dose of 50 e− /Å^2, and target defocus range between 0.5 and 1.2 µm. For TASK-2_{L127N} at pH 6.5, TASK-2_{WT} with bupivacaine at pH 8.5, and TASK-2_{WT} with bupivacaine at pH 6.5, images were collected at a pixel size of 0.57 Å/px, total dose of 50 e− /Å^2, and a target defocus range between 0.5 and 1.2 µm. See Table S1 for complete data collection statistics.

Cryo-EM data processing

For TASK-2$_{WT}$ in nanodiscs at pH 6.5, two datasets were collected. For collection 1, 4,143 movies were motion-corrected and binned with MotionCor2 in RELION v3.1 and contrast transfer function (CTF) parameters were fit with CTFFIND4.1[2,3]. Template-free auto-picking was performed on 5 micrographs using a Laplacian of Gaussian (LoG) picker. 1,066 particles were used for template picking on 2,889 movies CTF fit to 5.0 Å or better, yielding 1,456,265 particles which were extracted at 0.727 Å/px with a box size of 174 Å. 3 rounds of 2D classification yielded a cleaned particle stack of 118,873 particles that were used for TOPAZ training[4]. TOPAZ training and extraction yielded 1,706,965 particles which were extracted at 1.454 Å/px with a box size of 174 Å. Iterative rounds of 3D classification yielded 61,210 particles which were unbinned and used for Bayesian polishing in RELION v3.1

For collection 2, 2,488 movies were motion-corrected and binned with MotionCor2 in RELION v3.1 and contrast transfer function (CTF) parameters were fit with CTFFIND4.1[2,3]. Template-free auto-picking was performed on 5 micrographs using a Laplacian of Gaussian (LoG) picker. 3,456 particles were used for template picking on 1,440 movies CTF fit to 5.0 Å or better, yielding 373,748 particles which were extracted at 0.727 Å/px with a box size of 174 Å. 3 rounds of 2D classification yielded a cleaned particle stack of 55,345 particles that were used for TOPAZ training[4]. TOPAZ training and extraction yielded 677,865 particles which were extracted at 1.454 Å/px with a box size of 174 Å. Iterative rounds of 3D classification yielded 65,815 particles which were unbinned and used for Bayesian polishing in RELION v3.1. Polished particles were subjected to 1 round of 2D classification before a final *ab-initio.* A final non-uniform refinement yielded our final 2.64 Å map (C1, 110,525 particles).

For TASK-2$_{L127N}$ in nanodiscs at pH 6.5, 5,557 movies were motion-corrected with MotionCor2 in RELION v3.1 and contrast transfer function (CTF) parameters were fit with CTFFIND4.1[2,3]. Template-free auto-picking was performed using a Laplacian of Gaussian (LoG) picker on 5,003 micrographs CTF fit to 5.0 Å or better, yielding 1,222,677 particles which were extracted at 0.57 Å/px with a box size of 182 Å. 7 rounds of 2D classification were done in Relion v3.1 yielding 125,096 particles which were moved to cryoSPARC v3.3.1[5]. Iterative rounds of *ab-initio*s and 2D classification yielded a cleaned particle stack of 26,420 particles that were used for TOPAZ training[4]. TOPAZ training and extraction yielded 1,468,093 particles which were extracted at 1.14 Å/px with a box size of 205 Å. 3 rounds of classification in RELION v3.1 yielded 333,275 particles which were re-extracted at 0.57 Å/px and a box size of 205 Å and were transferred to cryoSPARC v3.3.1. Iterative rounds of *ab-initio* and 2D classification yielded 54,997 particles. A preliminary *ab-initio* and non-uniform refinement yielded a 3.00 Å map which was subsequently used for Bayesian polishing in RELION v3.1. Polished particles were subjected to 1 round of 2D classification before a final *ab-initio.* A final non-uniform refinement yielded our final 2.75 Å map (C1, 51,195 particles).

For TASK-2$_{K245A}$ at pH 6.5, 8,364 movies were motion-corrected with MotionCor2 in Relion v3.1 and contrast transfer function (CTF) parameters were fit with CTFFIND4.1[2,3]. Template-free auto-picking was performed on 6 micrographs using a Laplacian of Gaussian (LoG) picker. 6,593 particles were used for template picking on 5,784 movies CTF fit to 5.0 Å or better, yielding 5,882,461 particles which were extracted at 2.23 Å/px with a box size of 200 Å. 3 rounds of 2D classification were done in Relion v3.1 yielding 780,345 particles which

were re-extracted at 1.115 Å/px and moved to cryoSPARC v3.3.1[5]. Iterative rounds of *ab-initio*s and 2D classification yielded a cleaned particle stack of 97,135 particles that were used for TOPAZ training[4], yielding 1,788,989 particles which were extracted at 1.115 Å/px with a box size of 182 Å. Iterative rounds of *ab-initios*, heterogenous refinement, and 2D classification yielded a particle stack of 316,544 particles. A preliminary *ab-initio* and non-uniform refinement yielded a 3.60 Å map which was subsequently used for Bayesian polishing in Relion 4.1. Polished particles were subjected to 1 round of 2D classification before a final *ab-initio,* local CTF refinement, and global CTF refinement. A final non-uniform refinement yielded our final 3.22 Å map (C1, 310,671 particles).

For TASK-2_{WT} at pH 8.5 in the presence of bupivacaine, 4,969 movies were motion-corrected with MotionCor2 in Relion v3.1 and contrast transfer function (CTF) parameters were fit with CTFFIND4.1[2,3]. Template-free auto-picking was performed on 10 micrographs using a Laplacian of Gaussian (LoG) picker. 3,054 particles were used for template picking on 4,807 movies CTF fit to 5.0 or better, yielding 1,360,838 particles which were extracted at 0.57 Å/px with a box size of 182 Å. 4 rounds of 2D classification were done in Relion v3.1 yielding 230,977 particles that were used for TOPAZ training[4]. TOPAZ training and extraction yielded 1,320,431 particles which were extracted at 0.57 Å/px with a box size of 182 Å. 4 rounds of 2D classification were done in Relion v3.1, yielding 351,548 particles which were moved to cryoSPARC v3.3.1[5]. Iterative rounds of 2D classification and *ab-initio* were conducted, leaving 38,313 particles. A preliminary *ab-initio* and non-uniform refinement yielded a 3.03 Å map which was subsequently used for Bayesian polishing in Relion v3.1. Polished particles were subjected to 1 round of 2D classification before a final *ab-initio*. A final non-uniform refinement yielded our final 2.99 Å map (C1, 29,149 particles).

For TASK-2_{WT} at pH 6.5 in the presence of bupivacaine, 5,016 movies were motion-corrected with MotionCor2 in Relion v3.1 and contrast transfer function (CTF) parameters were fit with CTFFIND4.1[2,3]. Template-free auto-picking was performed on 11 micrographs using a Laplacian of Gaussian (LoG) picker. 2,291 particles were used for template picking on 4,918 movies CTF fit to 5.0 or better, yielding 977,170 particles which were extracted at 0.57 Å/px with a box size of 182 Å. 4 rounds of 2D classification were done in Relion v3.1 yielding 115,224 particles that were used for TOPAZ training[4]. TOPAZ training and extraction yielded 1,030,597 particles which were extracted at 1.14 Å/px with a box size of 182 and moved to cryoSPARC v3.3.1[5]. Iterative rounds of *ab-initios*, heterogenous refinement, and 2D classification yielded a particle stack of 126,792 particles. A preliminary *ab-initio* and non-uniform refinement yielded a 3.09 Å map which was subsequently used for Bayesian polishing in Relion 3.1. Polished particles were subjected to 1 round of 2D classification before a final 2-class *ab-initio*. A final non-uniform refinement yielded our final 2.91 Å map (C1, 105,900 particles).

Modeling and Refinement

The final non-uniformed local refined and sharpened maps from Cryosparc were used for modeling. An initial TASK-2 model (PDB: 6WLV for low pH structures and 6WM0 for high pH structures[1]) was rigid body fit to the density in Phenix[6]. Model building was performed iteratively with manual adjustment in Coot[7], global real space refinement in Phenix[6], and geometry assessment in Phenix[8] and Molprobity[9].

Electrophysiology

For patch clamp recording from *Xenopus* oocytes, a gene encoding full-length *Mus musculus* TASK-2 (UniProt Q9JK62) was codon optimized for eukaryotic expression, synthesized (Genewiz), and cloned into a modified pGEMHE vector using Xho1 and EcoR1 restriction sites. The transcribed messages encode *Mus musculus* TASK-2 (KCNK5) with three additional amino acids (SNS) at the C-terminus. TASK-2 mutants were introduced by polymerase chain reaction. Linearized DNA was transcribed *in vitro* using T7 polymerase. Complementary RNA (0.1 to 1 ng for single channel recordings, 1.5-3 ng for two-electrode voltage clamp recordings, or 10 ng for macroscopic patch recordings) in 50 nL H2O was injected into *Xenopus laevis* oocytes extracted from anesthetized frogs.

All patch recordings were performed in the inside-out configuration at 25 °C. Patches were excised from oocytes 1 to 5 d after RNA injection. Recordings were made once currents reached a magnitude suitable for each experiment. Neither the interval between injection and recording nor the region of the oocyte from which patches were excised was matched across constructs. Borosilicate glass pipettes were pulled to 2-5 MΩ resistance. Patches were formed using a high-speed pressure clamp device (HSPC-2-SB, ALA Scientific Instruments). Recordings were performed in a ten-fold [K^+] gradient with a pipette solution containing 15 mM KCl, 135 mM NaCl and 10 mM HEPES (pH 6.2, 6.5, 7.2, 7.5, 9.1, 9.5 with NaOH) and a bath solution containing 150 mM KCl and 10 mM HEPES (pH 6.5, 7.5, 9.5 with KOH). Currents were recorded using an Axopatch 200B amplifier (Molecular Devices) at a bandwidth of 1 kHz and digitized with an Digidata 1550B (Molecular Devices) at 50 kHz using pClamp10.7 software. Patches were first evaluated with voltage-step protocols to confirm TASK-2 expression and minimal non-specific leak based on reversal potential near E_{K+}. All presented data were recorded from a holding potential of 0 mV to minimize contribution from non-specific leak. Aggregated data were from patches from n ≥ 2 different cells on different days. Similar currents were not observed in control uninjected oocytes.

For single-channel recordings, each patch was subjected to ≥ 3 bath exchanges to sample different pH_{int} values under constant pH_{ext} defined by the pipette solution. For analysis, recordings were separated into segments according to pH_{int}. To maintain the integrity of brief TASK-2 dwell times, records were not further filtered unless explicitly stated. Baseline correction was performed manually based on the observable closed state for each recording to account for minor baseline drift that occurred during some solution exchanges. Single channel open-close transitions were idealized by half-amplitude threshold crossing using previously reported software[10] or Clampfit 10.7 with a 0.5 pA event detection cutoff. Dwell time event lists were used to calculate dwell time histograms and open probabilities. Multiple Gaussian fits to dwell time histograms were calculated in GraphPad Prism. Amplitude histograms were generated from baseline corrected records in Clampfit 10.7. Open event amplitude histograms were generated from fit events lists after removing all closed events. Histograms were plotted in Prism using a bin size of 0.05 pA (Fig. S12). For some records, all event data were reduced by a factor of 10 prior to plotting amplitude histograms.

For macroscopic inside-out recordings, patches were included in dose-response analysis only when currents were clearly distinguishable from baseline noise at all tested pH values. Patches with smaller currents were excluded. Because channel number, patch area, and oocyte batch were not controlled between constructs,

maximal current amplitudes are not comparable across constructs, in either these recordings (Table S3) or the whole-cell recordings below (Table S4). pH sensitivity was derived from currents normalized within each patch or cell and is therefore independent of these factors.

For two-electrode voltage clamp recording, pipette solution contained 3 M KCl and bath solution contained 96 mM NaCl, 2 mM KCl, 1.8 mM CaCl2 , 1 mM $MgCl_2$, 2.5 mM sodium pyruvate, and 20 mM buffer to vary pH_{ext} (MES pH 5.5, 6.0, and 6.5 or HEPES pH 7.0, 7.5, 8.0, or 8.5). Bath volume was kept at ~300 uL during recording. Eggs were initially placed in the recording chamber in pH_{ext} 8.5 and then perfused to vary pH_{ext}. Currents were recorded and low pass filtered at 2 kHz using an Axon Instruments Axoclamp 900A amplifier and digitized at 10 kHz with a Sutter Dendrite digitizer. Voltage families were recorded with 200 ms steps ranging from -100 to +40 mV to confirm K^+-selective currents. The effects of pH_{ext} on channel activity were quantified by the reduction in channel current evoked at 0 mV. Currents were normalized to those recorded at pH_{ext} 8.5 to facilitate comparisons across cells. Dose responses were fit with four-parametric Boltzmann curves to aggregated data.

Quantification And Statistical Analysis

Data analysis was performed using Clampfit 10.7, Microsoft Excel, and Graphpad Prism. All data are reported as mean ± SEM with n representing number of patches, cells, or particles. Statistical tests were made with ordinary one-way ANOVA with Dunnett's corrections for multiple comparisons or unpaired T-tests in Prism software.

Animals

Oocytes were harvested from female *Xenopus laevis* frogs obtained from Nasco and housed at the University of California, Berkeley in the Office for Animal Care and Use (OLAC) facilities. The use of oocytes was approved by the Animal Use and Care Committee at the University of California, Berkeley under AUP-2019-11-12743.

Table S1 Cryo-EM data collection, refinement, and validation statistics

	TASK-2 pH 6.5<br><br>EMDB-75365<br>PDB 10OY	TASK-2 L127N pH 6.5<br><br>EMDB-75367<br>PDB 10PB	TASK-2 pH 8.5 + Bupivacaine<br><br>EMDB-75371<br>PDB 10PF	TASK-2 pH 6.5 + Bupivacaine<br><br>EMDB-75368<br>PDB 10PC	TASK-2 K245A pH 6.5<br><br>EMDB-75369<br>PDB 10PD
**Data collection and processing**					
Voltage (kV)	300	300	300	300	200
Electron exposure (e–/Å^2)	50	50	50	50	50
Target defocus range (μm)	–0.5 to –1.2	–0.5 to –1.2	–0.5 to –1.2	–0.5 to –1.2	–0.7 to –2.2
Pixel size (Å)	0.3635	0.57	0.57	0.57	1.115
Initial movies (no.)	6531	5516	4969	5016	8364
Final movies (no.)	4329	5003	4807	4918	5784
Symmetry imposed	C1	C1	C1	C1	C1
Initial particle images (no.)	2,384,830	1,468,093	1,320,431	1,030,597	1,788,989
Final particle images (no.)	110,525	51,195	29,149	105,900	310,671
Map resolution (Å)	2.64	2.75	2.99	2.91	3.22
FSC threshold	0.143	0.143	0.143	0.143	0.143
**Refinement**					
Model resolution (Å)	2.6	2.7	3.0	2.9	3.3
FSC threshold	0.143	0.143	0.143	0.143	0.143
Map sharpening *B* factor (Å^2)	-82.5	-75.9	-82.1	-111.4	-168.2
Model composition					
Non-hydrogen atoms	4679	4492	4577	4558	4202
Protein residues	538	527	539	538	511
Ligands	26	23	19	20	13
K	3	3	4	3	3
PEE	24	20	14	17	16
Bupivacaine	0	0	1	0	0
Water	21	31	21	19	0
B factors (mean, Å^2)					
Protein	60.00	81.36	118.92	106.98	66.83
Ligand	63.97	81.80	99.10	94.73	68.21
Water	29.45	64.05	93.32	68.31	
R.m.s. deviations					
Bond lengths (Å)	0.006	0.006	0.004	0.007	0.003
Bond angles (°)	0.780	0.781	0.575	0.798	0.490
Validation					
MolProbity score	1.19	1.10	1.09	1.29	1.07
Clashscore	4.01	3.12	3.08	4.80	2.19
Rotamer favored (%)	97.53	96.11	96.65	97.76	96.91
Rotamer outliers (%)	0.67	0.92	0	1.12	0
Ramachandran plot					
Favored (%)	98.31	98.08	99.06	98.12	97.62
Allowed (%)	1.69	1.92	0.94	1.88	2.38
Disallowed (%)	0	0	0	0	0

Table S2: Summary data from single channel recordings

Construct	External pH	Internal pH	n	Total duration analyzed (s)	P_o	P value relative to $TASK_{WT}$ P_o
TASK-2_{WT}	6.5	6.2	3		nd	
		7.5	3		nd	
		9.5	3		nd	
	7.5	6.2	3		nd	
		7.2	3	475	0.01 ± 0.003	
		9.5	3	91	0.47 ± 0.09	
	9.1	6.2	3		nd	
		7.2	3	752	0.09 ± 0.03	
		9.5	3	540	0.39 ± 0.09	
TASK-2_{L127N}	6.5	6.5	3	152	0.03 ± 0.02	-
		7.5	3	262	0.08 ± 0.07	-
		9.5	3	151	0.24 ± 0.01	-
	7.5	6.2	3	148	0.22 ± 0.04	-
		7.2	3	280	0.34 ± 0.04	< 0.0001****
		9.5	3	152	0.50 ± 0.06	0.95
	9.1	6.2	3	1332	0.76 ± 0.11	-
		7.2	3	610	0.81 ± 0.02	< 0.0001****
		9.5	3	890	0.89 ± 0.02	0.0052**
TASK-2_{K245A}	7.5	6.5	3		nd	
		7.5	3	463	0.001 ± 0.0002	0.99
		9.5	3	167	0.03 ± 0.02	0.0033**
TASK-$2_{L127N/K245A}$	7.5	6.5	3	281	0.43 ± 0.03	
		7.5	3	280	0.39 ± 0.02	< 0.0001****
		9.5	3	319	0.47 ± 0.06	0.99

Comparisons made with one-way Anova with Dunnet correction for multiple comparisons or unpaired T-test.

Construct	n	$pH_{int\ 1/2}$	$pH_{1/2}$ p value	Hill slope	Fit R^2	I_{max} (pA) pH_{int} 9.5, 0 mV
Wild-type	6	8.05 ± 0.14	-	-1.08 ± 0.03	0.97	164 ± 21
TASK-2_{K245A}	6	7.80 ± 0.11	0.29	-0.69 ± 0.09	0.71	89 ± 32
TASK-2_{L127N}	5	7.94 ± 0.03	0.07	-0.86 ± 0.05	0.95	33 ± 8
TASK-$2_{L127N/K245A}$	3	8.19 ± 0.09	0.65	-0.81 ± 0.11	0.89	205 ± 2

Table S3: Summary of fits to macroscopic inside-out recordings with varying pHint (Fig. S7A). $pH_{int\ 1/2}$ and Hill slope values are derived from currents normalized within each patch and are independent of the number of channels in the record. I_{max} indicates the magnitude of the currents analyzed and is not comparable between constructs (see Methods). Recordings were made at $pH_{ext\ 7.5}$ and a holding potential of 0 mV in a ten-fold [K+] gradient. Comparisons made with one-way ANOVA with Dunnett correction for multiple comparisons.

Construct	n	$pH_{ext\ 1/2}$	$pH_{1/2}$ p value	Hill slope	Fit R^2	I_{max} (µA) pH_{ext} 8.5, 0 mV
Wild-type	3	7.41 ± 0.03	-	-1.41± 0.13	0.96	5.10 ± 0.79
TASK-2_{K245A}	3	7.50 ± 0.01	0.29	-1.99 ± 0.08	0.96	5.16 ± 1.28
TASK-2_{L127N}	4	7.84 ± 0.01	0.02*	-1.61 ± 0.04	0.99	5.42 ± 0.15
TASK-$2_{L127N/K245A}$	3	7.18 ± 0.02	0.03*	-1.22 ± 0.06	0.99	2.36 ± 0.77

Table S4: Summary of fits to macroscopic whole-cell TEVC recordings with varying pHext (Fig. S7D). $pH_{ext\ 1/2}$ and Hill slope values are derived from currents normalized within each cell and are independent of the number of channels expressed. I_{max} indicates the magnitude of the currents analyzed and is not comparable between constructs (see Methods). Oocyte pH (pH_{int}) was not controlled in these recordings. Comparisons made with one-way ANOVA with Dunnett correction for multiple comparisons.

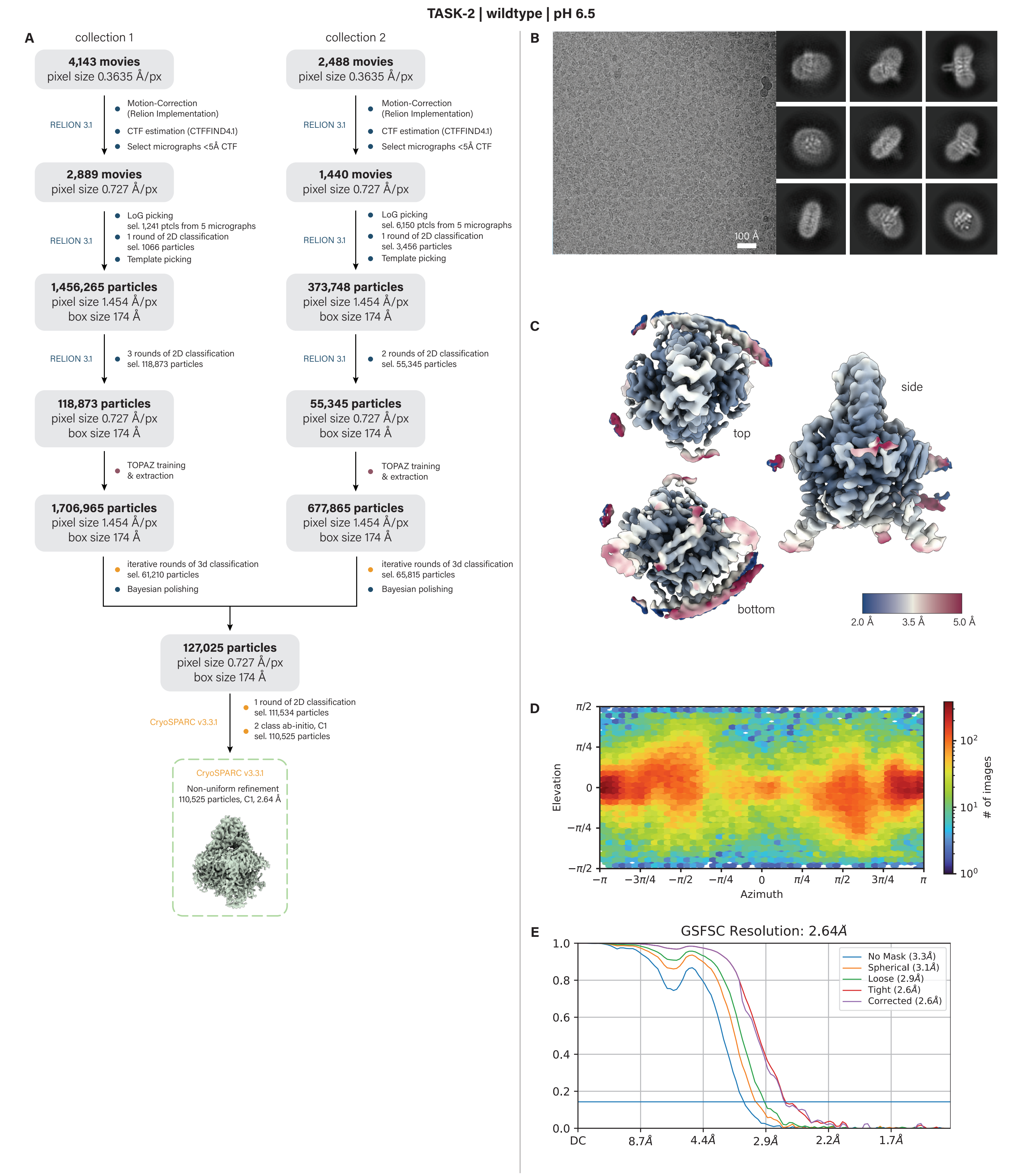

Figure S1. TASK-2$_{WT}$ pH 6.5 cryo-EM data processing and validation.
(A) Cryo-EM data processing pipeline and reconstructed map from final particle stack for TASK-2$_{WT}$ pH 6.5. (B) Example micrograph and 2D class averages. (C) Final sharpened map colored by local resolution and viewed from the membrane plane, top, and bottom. (D) View angle distribution of final particle stack. (E) Fourier shell correlation (FSC) versus resolution between half maps from final refinement.

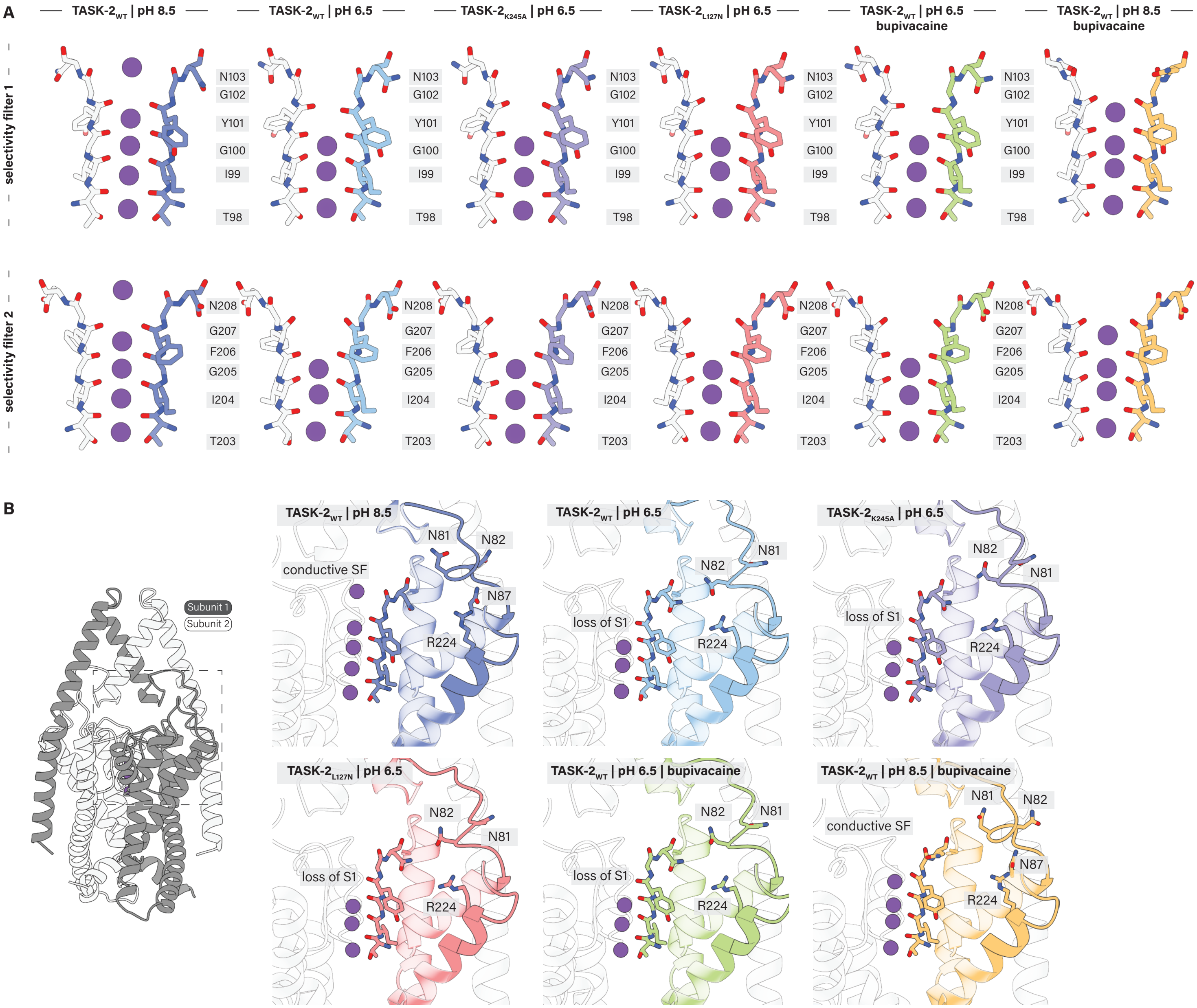

Figure S2. Comparison of extracellular gate in TASK-2 structures.
(A) Configuration of SF1 (residues 98-103, top) and SF2 (residues 203-208, bottom) in TASK-2 structures. (B) Comparison of external gate configuration in TASK-2 structures highlighting residues N82, N87, and R224. Model colors are as follows: TASK-2_{WT} at pH 8.5 (6WM0, navy), TASK-2_{WT} at pH 6.5 (light blue), TASK-2_{K245A} at pH 6.5 (purple), TASK-2_{L127N} at pH 6.5 (red),TASK-2_{WT} with bupivacaine at pH 6.5 (green), and TASK-2_{WT} with bupivacaine at pH 8.5 (orange).

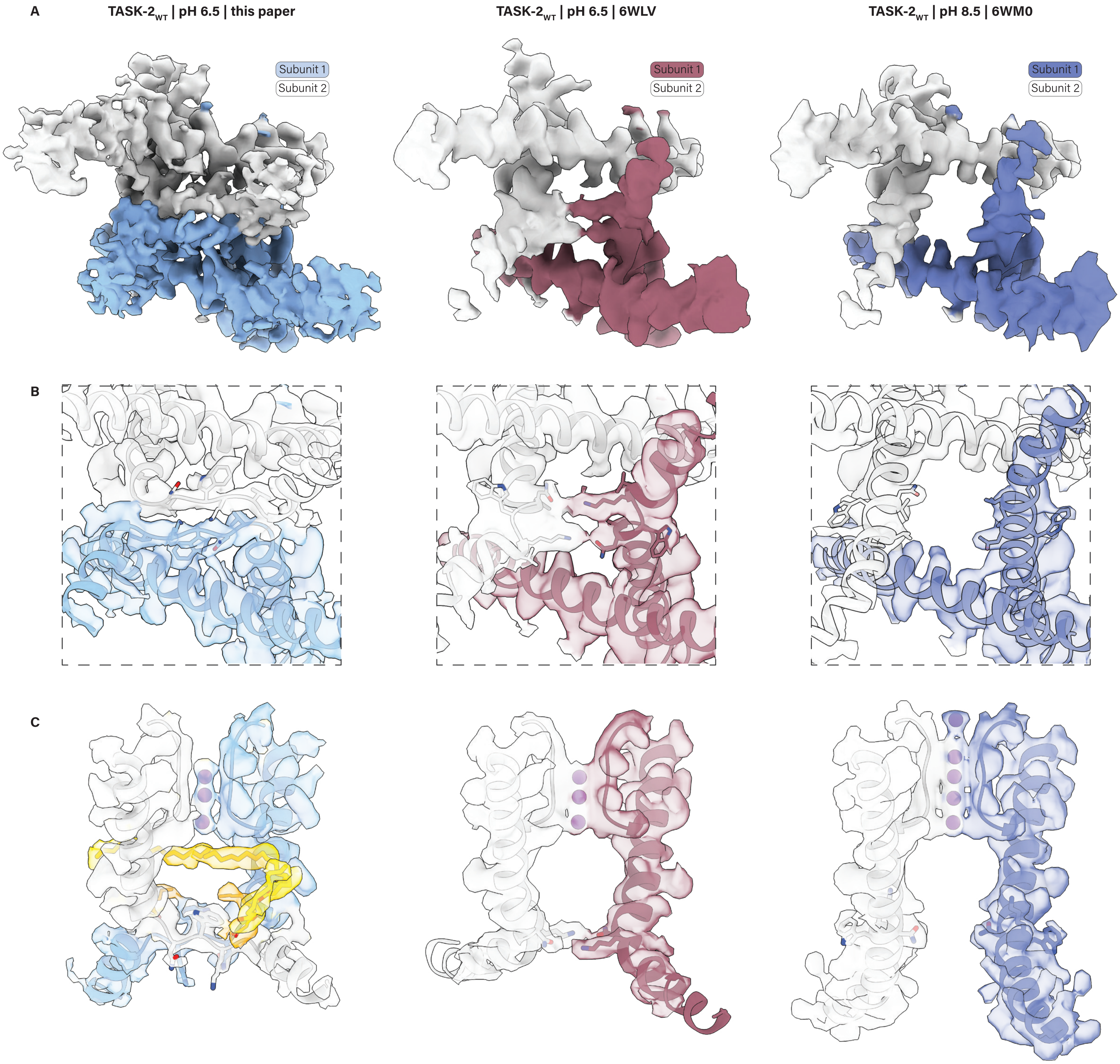

Figure S3. Comparison of TASK-2 cryo-EM maps and models.
(A) Cytosolic views of model carved density for 2.6 Å low pH structure reported here (light blue/white), previously reported 3.5 Å low pH structure (PDB: 6WLV) (maroon/white), and previously reported 3.5 Å high pH structure (PDB:6WM0) (navy/white) showing intracellular regions of TM2 and TM4. **(B)** Zoomed view of the intracellular face of the channel with residues N243, W244, and K245 shown as sticks. **(C)** Side views highlighting lipid densities and improved resolution around the Z-gate. A non-domain swapped conformation for TM4s was modeled into the previous low resolution low pH structure. Z-gate conformation and TM4 domain swap are clearly resolved in the improved map.

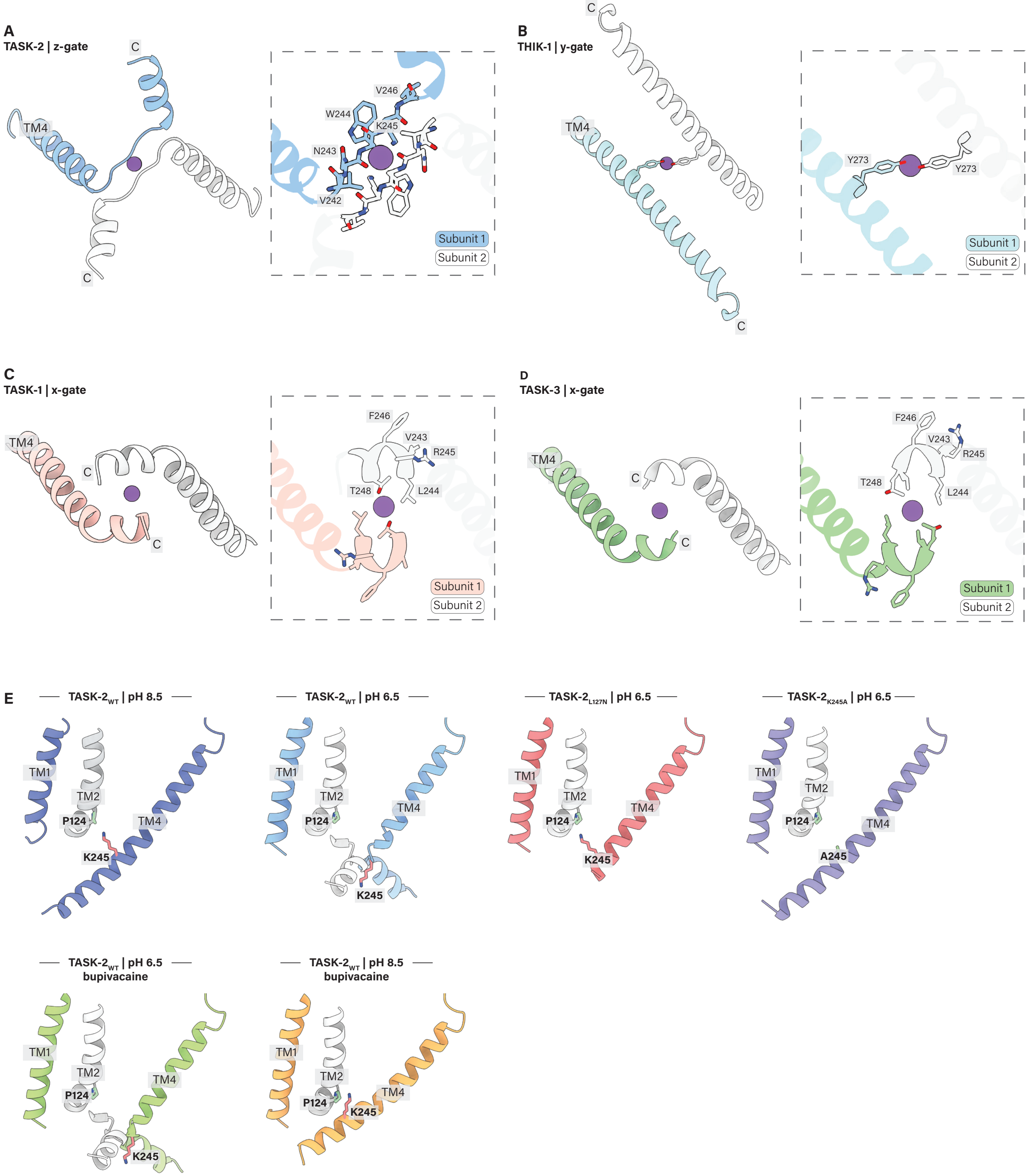

Figure S4. Comparison of internal protein gates in K2P channels.
(A) The Z-gate in TASK-2 formed by [242]VNWLV[246]. (B) The Y-gate in THIK-1 is formed by Y273. (C) The X-gate in TASK-1 formed by [243]VLRFMT[248]. (D) The X-gate in TASK-3 formed by [243]VLRFLT[248]. Cytosolic view of each TM4 and relative position of S4 potassium (left) and a zoomed view of associated internal gating residues (right). (E) Comparison of TM4 position between models. Models with closed zipper gates display both halves of the zipper gate. For (E), model colors are as follows: TASK-2_{WT} at pH 8.5 (6WM0, navy), TASK-2_{WT} at pH 6.5 (light blue), TASK-2_{K245A} at pH 6.5 (purple), TASK-2_{L127N} at pH 6.5 (red),TASK-2_{WT} with bupivacaine at pH 6.5 (green), and TASK-2_{WT} with bupivacaine at pH 8.5 (orange).

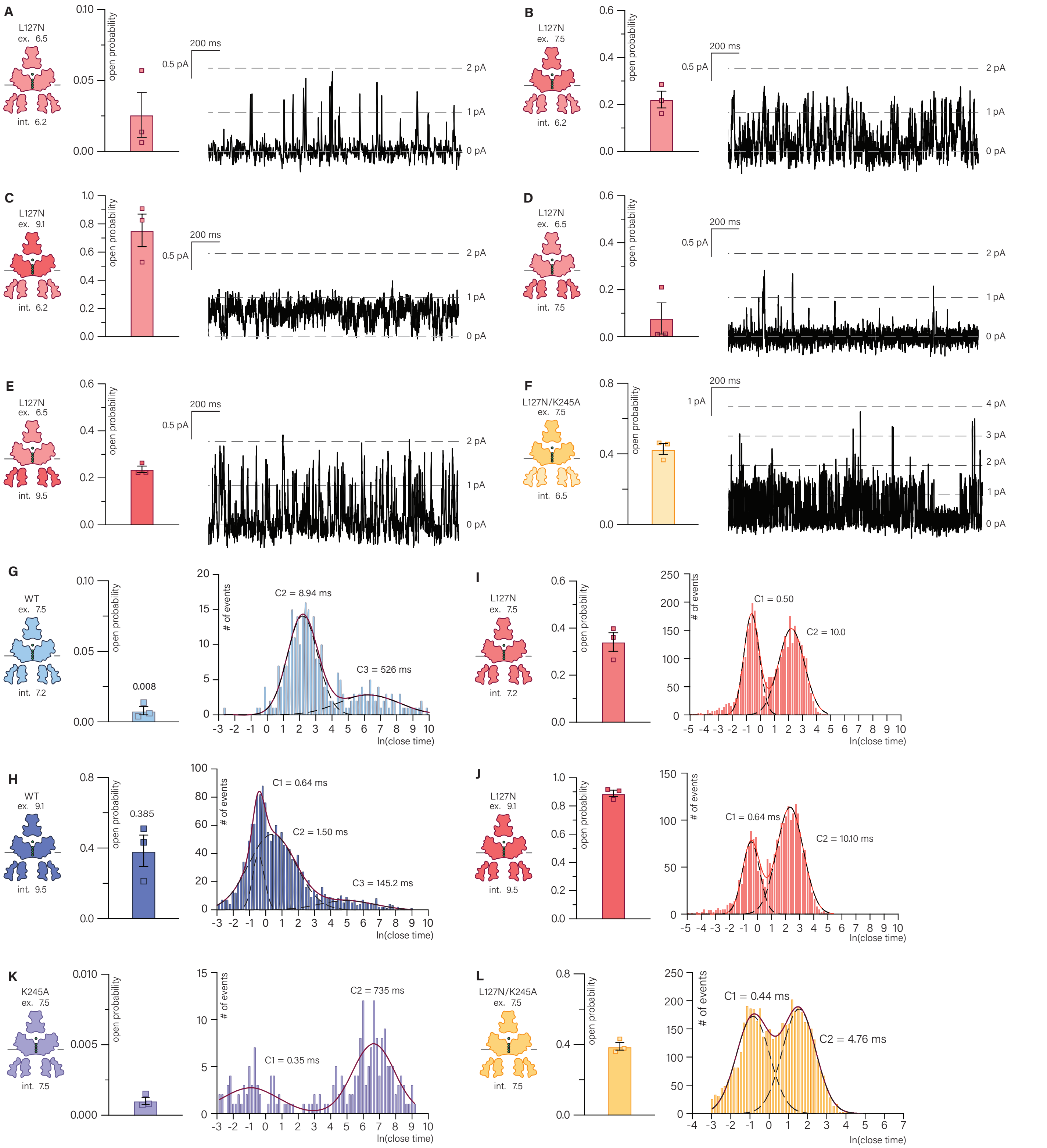

Figure S5. Representative single channel recordings and dwell time analysis.
(A) Open probability calculated from TASK-2$_{L127N}$ at acidic pH$_{int}$ and pH$_{ext}$ with accompanying 1.5s example traces. (P_o = 0.03 ± 0.02, n= 3).
(B) Open probability calculated from TASK-2$_{L127N}$ at acidic pH$_{int}$ and neutral pH$_{ext}$ with accompanying 1.5s example traces. (P_o = 0.22 ± 0.04, n= 3).
(C) Open probability calculated from TASK-2$_{L127N}$ at acidic pH$_{int}$ and alkaline pH$_{ext}$ with accompanying 1.5s example traces. (P_o = 0.76 ± 0.11, n= 3).
(D) Open probability calculated from TASK-2$_{L127N}$ at neutral pH$_{int}$ and acidic pH$_{ext}$ with accompanying 1.5s example traces. (P_o = 0.08 ± 0.07, n= 3).
(E) Open probability calculated from TASK-2$_{L127N}$ at alkaline pH$_{int}$ and acidic pH$_{ext}$ with accompanying 1.5s example traces. (P_o = 0.24 ± 0.01, n= 3).
(F) Open probability calculated from TASK-2$_{L127N/K245A}$ at acidic pH$_{int}$ and neutral pH$_{ext}$ with accompanying 1.5s example traces. (P_o = 0.43 ± 0.03, n= 3).
(G) example closed dwell time histogram for TASK-2$_{WT}$ at neutral pH$_{int}$ and pH$_{ext}$ (C2 = 8.94 ms, C3= 526 ms).
(H) example closed dwell time histogram for TASK-2$_{WT}$ at alkaline pH$_{int}$ and pH$_{ext}$ (C1 = 0.64 ms, C2= 1.5 ms, C3 = 145.2 ms).
(I) example closed dwell time histogram for TASK-2$_{L127N}$ at neutral pH$_{int}$ and pH$_{ext}$ (C1 = 0.50 ms, C2= 10.0 ms).
(J) example closed dwell time histogram for TASK-2$_{L127N}$ at alkaline pH$_{int}$ and pH$_{ext}$ (C1 = 0.64 ms, C2= 10.1 ms).
(K) example closed dwell time histogram for TASK-2$_{K245A}$ at neutral pH$_{int}$ and pH$_{ext}$ (C1 = 0.35 ms, C2= 735 ms).
(L) example closed dwell time histogram for TASK-2$_{L127N/K245A}$ at neutral pH$_{int}$ and pH$_{ext}$ (C1 = 0.44 ms, C2= 4.76 ms).
All recordings were performed at a holding potential of 0 mV in a ten-fold [K^+] gradient.

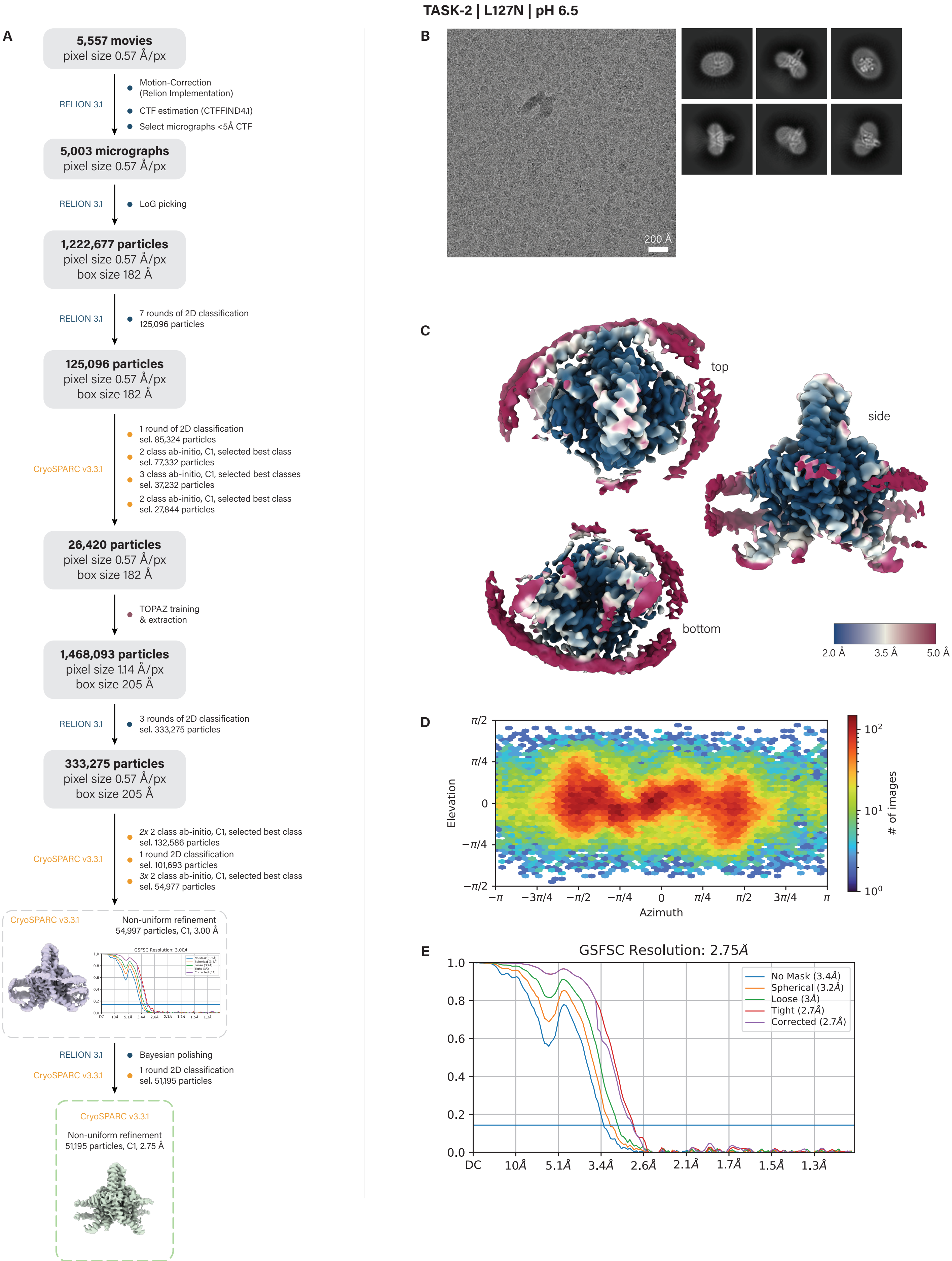

Figure S6. TASK-2$_{L127N}$ pH 6.5 cryo-EM data processing and validation.
(A) Cryo-EM data processing pipeline and reconstructed map from final particle stack for TASK-2$_{L127N}$ pH 6.5. (B) Example micrograph and 2D class averages. (C) Final sharpened map colored by local resolution and viewed from the membrane plane, top, and bottom. (D) View angle distribution of final particle stack. (E) Fourier shell correlation (FSC) versus resolution between half maps from final refinement.

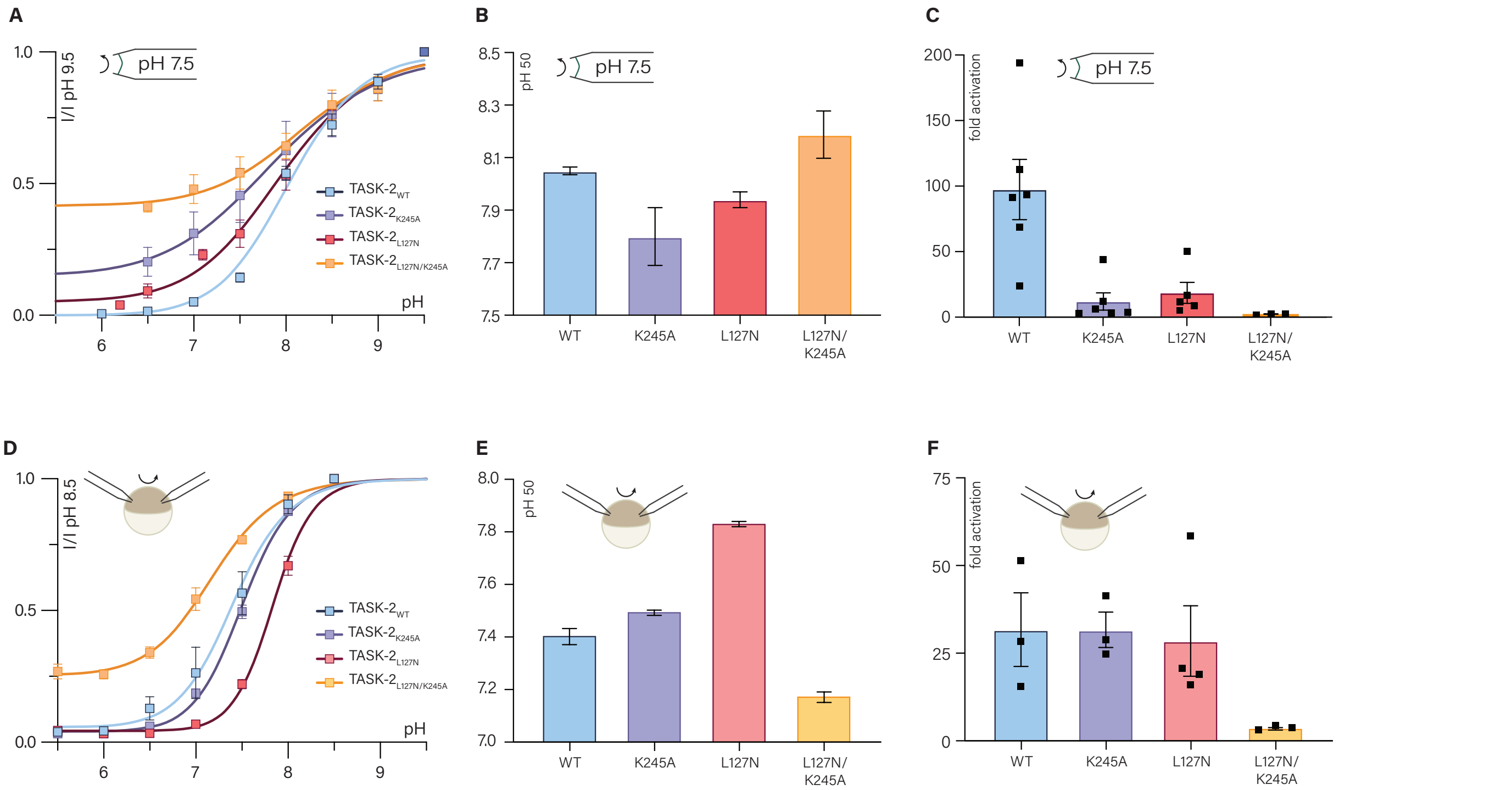

Figure S7. Macroscopic pH dependence of TASK-2 constructs.

(A) Dose response of macroscopic TASK-2 current to pH_{int} recorded from inside-out patches at pH_{ext} 7.5 and 0 mV. Currents are normalized to current at pH_{int} 9.5 for each patch and shown as mean ± SEM with Boltzmann fit ($pH_{1/2}$ = 8.05 ± 0.14, 7.80 ± 0.11, 7.94 ± 0.03, and 8.19 ± 0.09 for TASK-2_{WT}, TASK-2_{K245A}, TASK-2_{L127N}, and TASK-$2_{L127N/K245A}$; n = 6, 6, 5, and 3 patches, respectively). (B) $pH_{1/2}$ values from (A). Bars are mean ± SEM. (C) Fold activation of macroscopic current between pH_{int} 6.5 and pH_{int} 9.5. Bars are mean ± SEM with individual patches shown. (D) Dose response of macroscopic TASK-2 current to pH_{ext} recorded from oocytes by two-electrode voltage clamp. Currents are normalized to current at pH_{ext} 8.5 for each cell and shown as mean ± SEM with Boltzmann fits ($pH_{1/2}$ = 7.41 ± 0.03, 7.50 ± 0.01, 7.84 ± 0.01, and 7.18 ± 0.02 for TASK-2_{WT}, TASK-2_{K245A}, TASK-2_{L127N}, and TASK-$2_{L127N/K245A}$; n = 3, 3, 4, and 3 oocytes, respectively). (E) $pH_{1/2}$ values from (D). Bars are mean ± SEM. (F) Fold activation of macroscopic current between pH_{ext} 5.5 and pH_{ext} 8.5. Bars are mean ± SEM with individual oocytes shown. All recordings were made at a holding potential of 0 mV. Recordings in (A–C) were made from inside-out patches in a ten-fold [K^+] gradient; recordings in (D–F) were made from oocytes in 2 mM K^+ bath solutions.

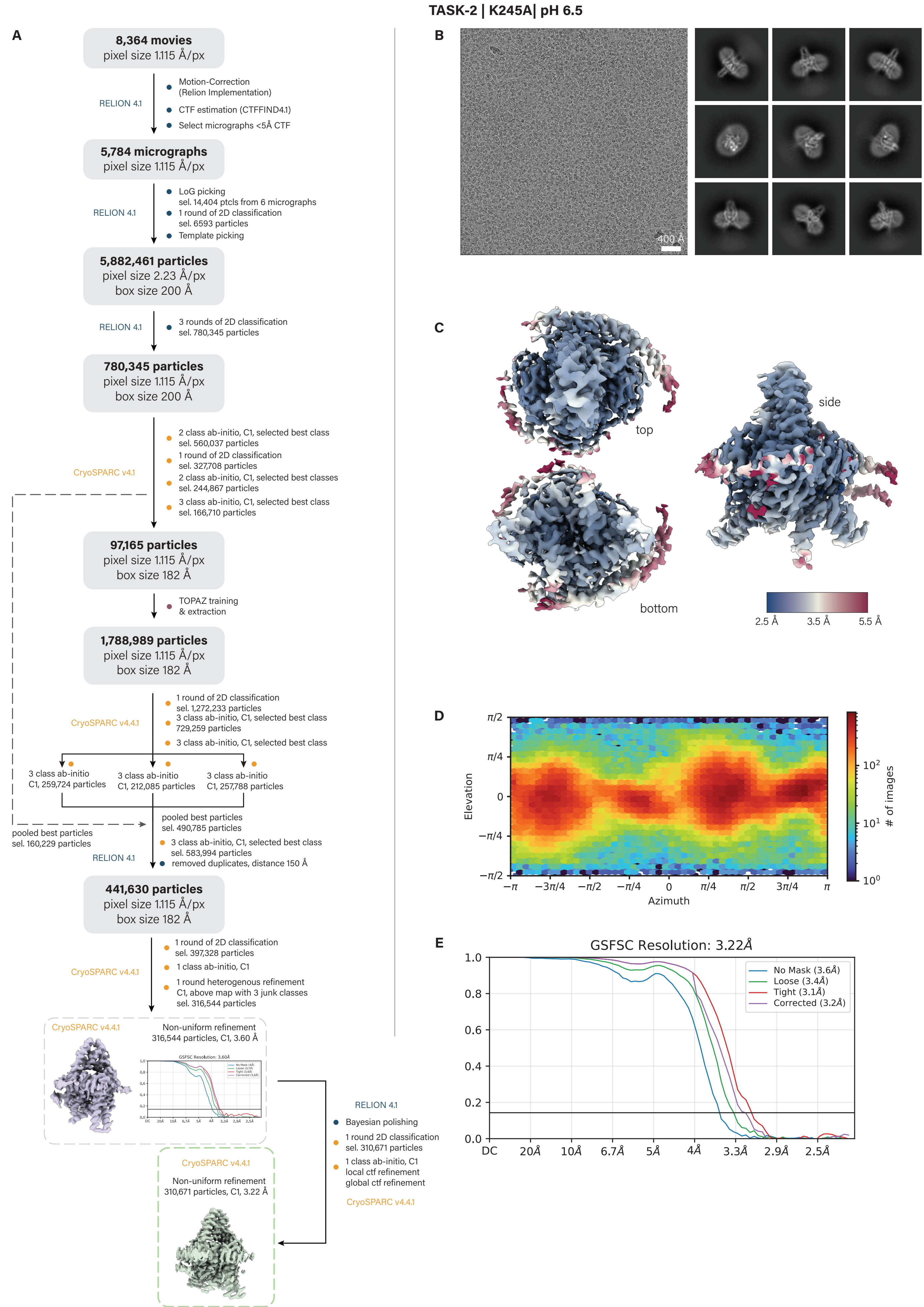

Figure S8. TASK-2$_{K245A}$ pH 6.5 cryo-EM data processing and validation.
(A) Cryo-EM data processing pipeline and reconstructed map from final particle stack for TASK-2$_{K245A}$ at pH 6.5. (B) Example micrograph and 2D class averages. (C) Final sharpened map colored by local resolution and viewed from the membrane plane, top, and bottom. (D) View angle distribution of final particle stack. (E) Fourier shell correlation (FSC) versus resolution between half maps from final refinement.

TASK-2 | bupivacaine| pH 8.5

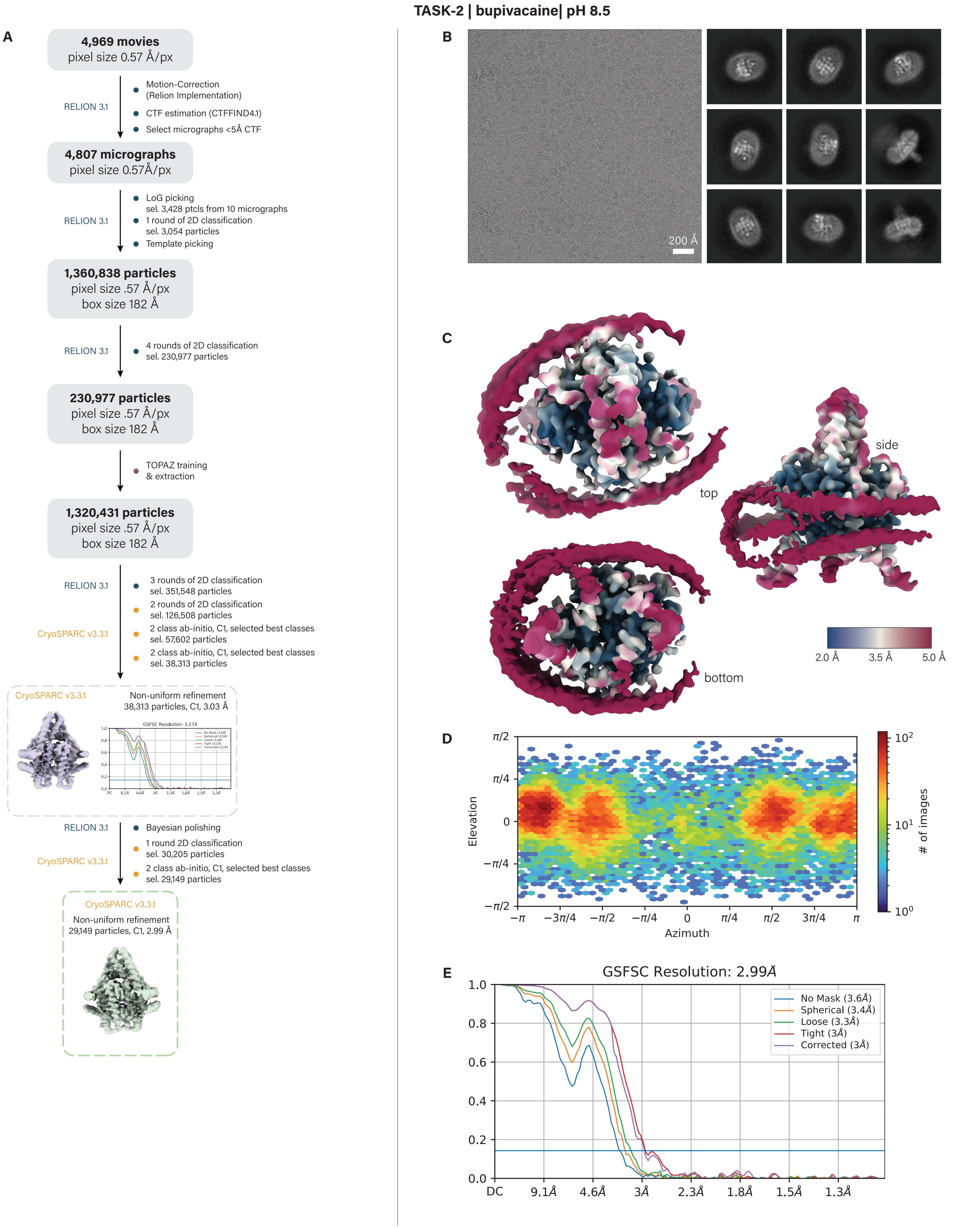

Figure S9. TASK-2$_{WT}$ pH 8.5 with bupivacaine cryo-EM data processing and validation.
(A) Cryo-EM data processing pipeline and reconstructed map from final particle stack for TASK-2$_{WT}$ at pH 8.5 in the presence of bupivacaine. (B) Example micrograph and 2D class averages. (C) Final sharpened map colored by local resolution and viewed from the membrane plane, top, and bottom. (D) View angle distribution of final particle stack. (E) Fourier shell correlation (FSC) versus resolution between half maps from final refinement.

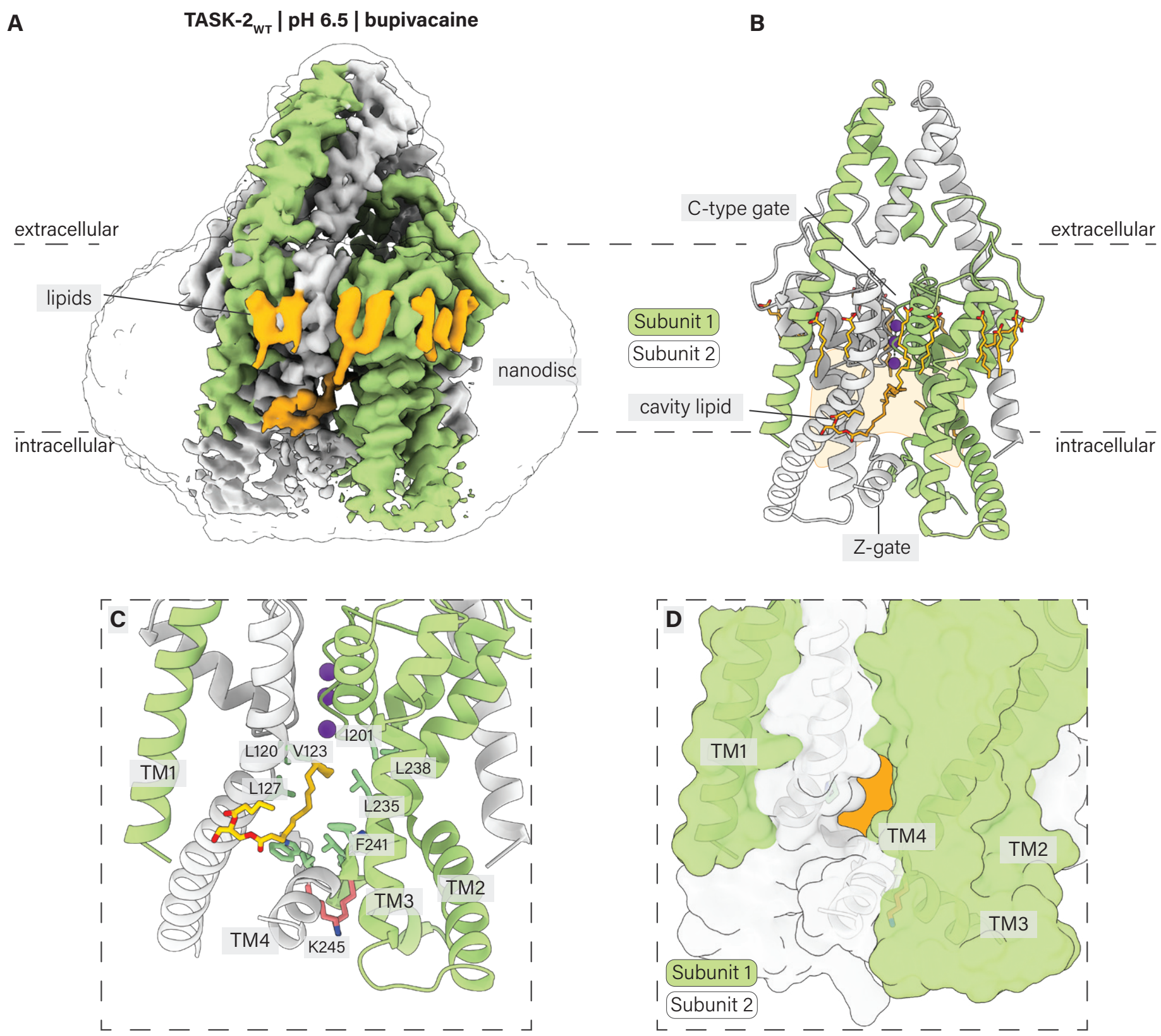

Figure S10. Lipids occupy the TASK-2$_{WT}$ channel cavity at pH 6.5 in the presence of bupivacaine. (A) Side view of TASK-2$_{WT}$ with bupivacaine pH 6.5 cryo-EM map and (B) non-conductive channel model. Channel subunits are green and white, K^+ is purple, and lipids are gold. Side views of (C) model and (D) protein surface highlighting lateral fenestration and cavity environment.

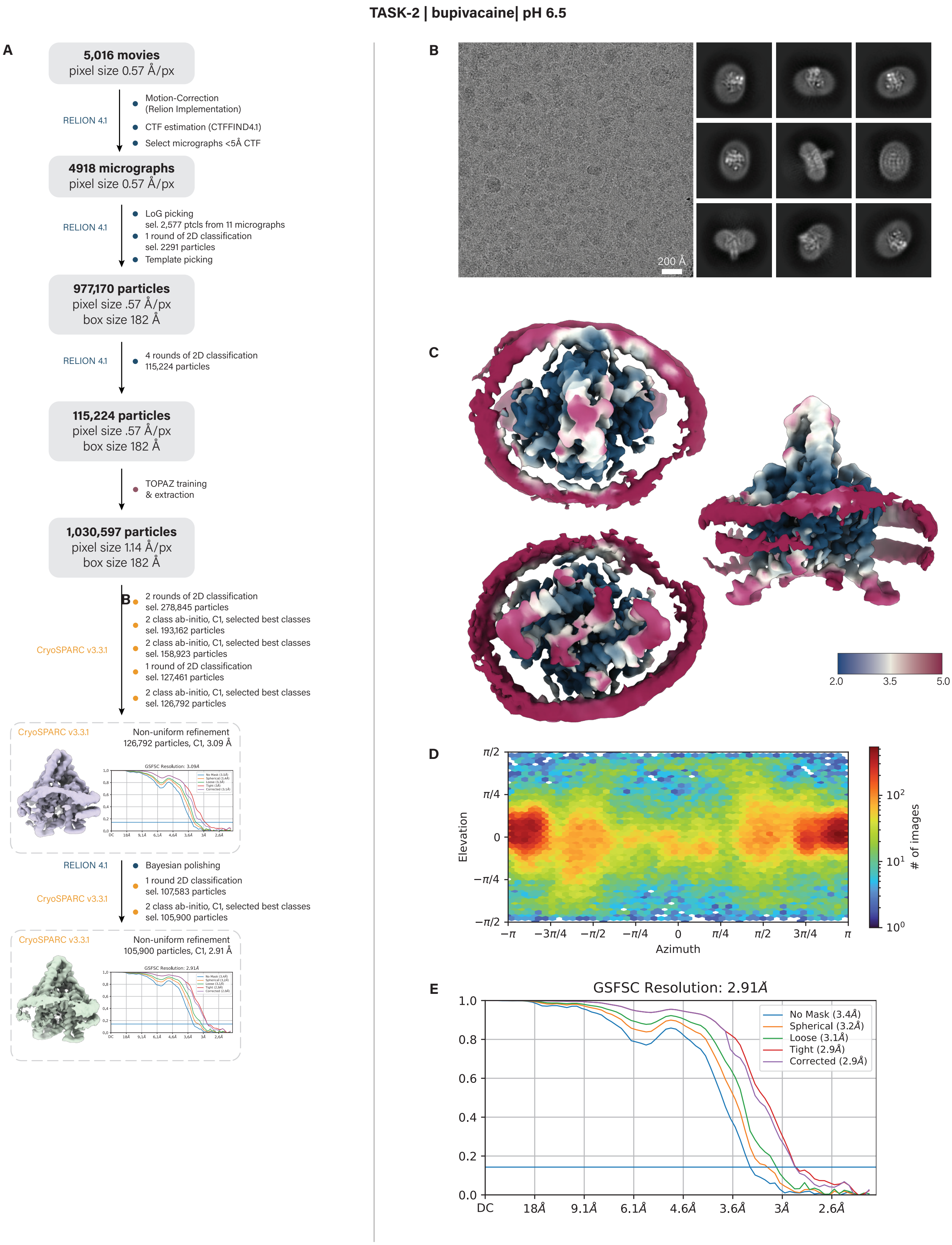

Figure S11. TASK-2$_{WT}$ pH 6.5 with bupivacaine cryo-EM data processing and validation.
(A) Cryo-EM data processing pipeline and reconstructed map from final particle stack for TASK-2$_{WT}$ at pH 6.5 in the presence of bupivacaine. (B) Example micrograph and 2D class averages. (C) Final sharpened map colored by local resolution and viewed from the membrane plane, top, and bottom. (D) View angle distribution of final particle stack. (E) Fourier shell correlation (FSC) versus resolution between half maps from final refinement.

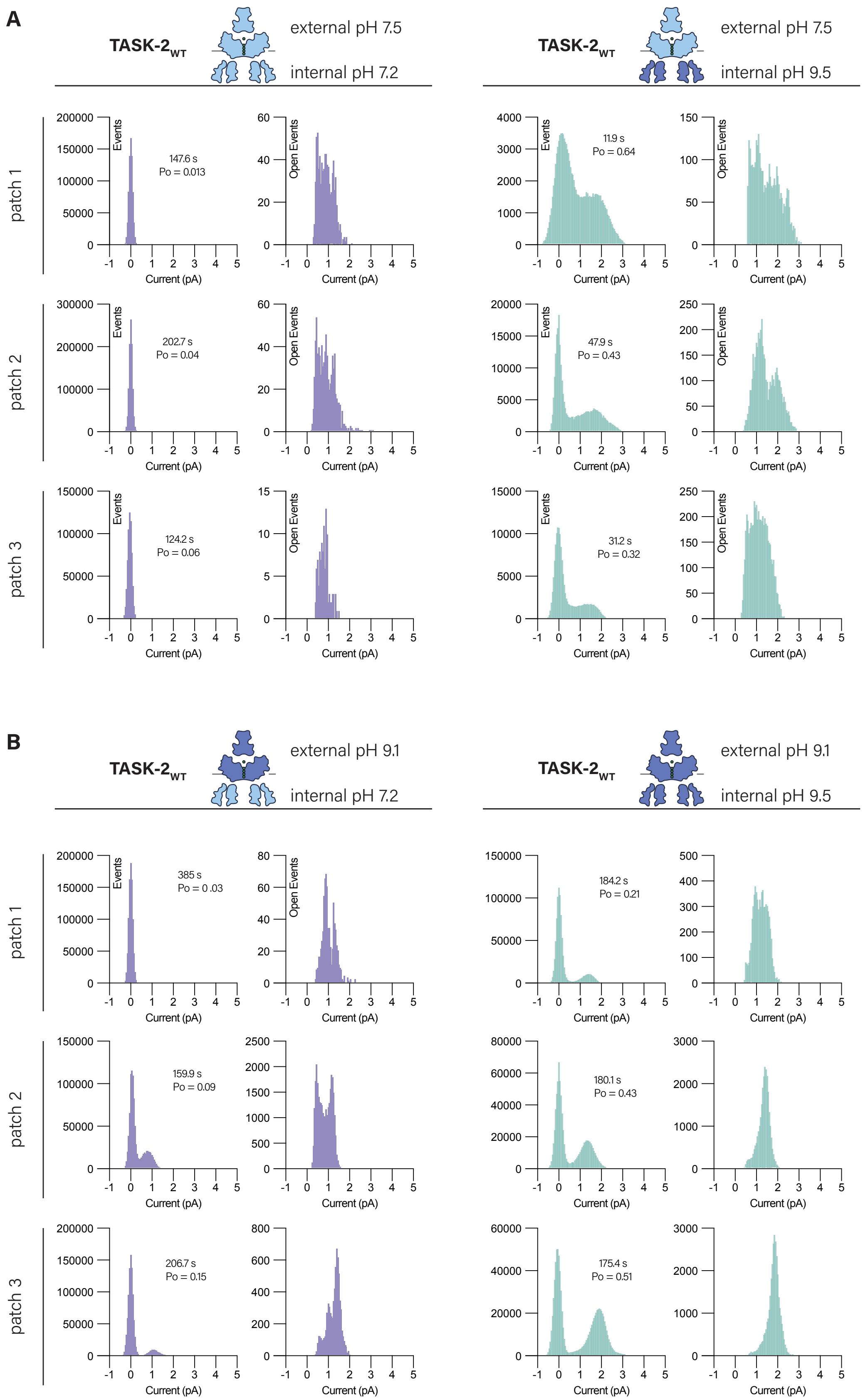
A
TASK-2WT
external pH 7.5
internal pH 7.2
TASK-2WT
external pH 7.5
internal pH 9.5
patch 1
patch 2
patch 3
Events
Open Events
Current (pA)
147.6 s
Po = 0.013
202.7 s
Po = 0.04
124.2 s
Po = 0.06
11.9 s
Po = 0.64
47.9 s
Po = 0.43
31.2 s
Po = 0.32
B
TASK-2WT
external pH 9.1
internal pH 7.2
TASK-2WT
external pH 9.1
internal pH 9.5
385 s
Po = 0.03
159.9 s
Po = 0.09
206.7 s
Po = 0.15
184.2 s
Po = 0.21
180.1 s
Po = 0.43
175.4 s
Po = 0.51

C

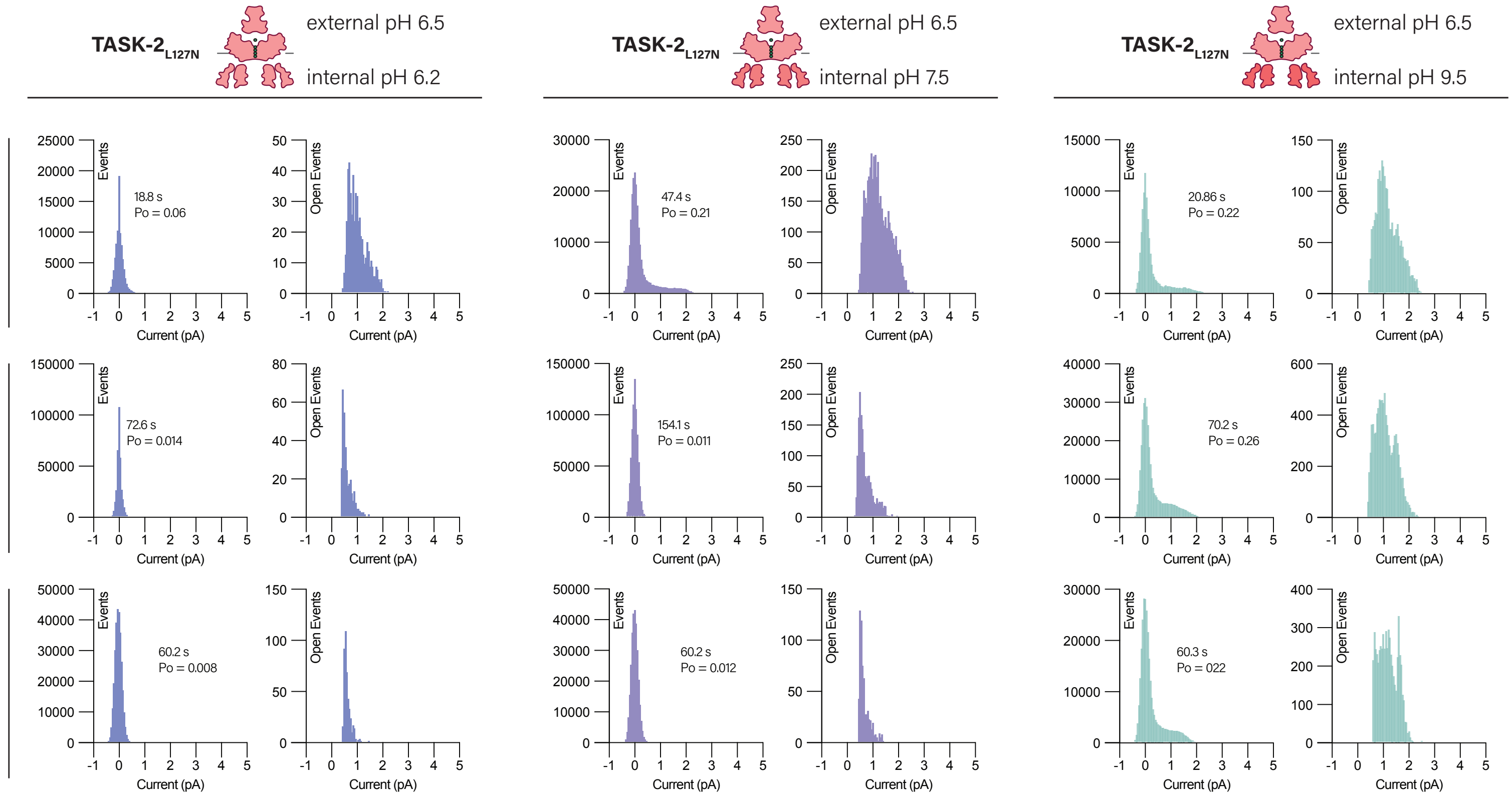
TASK-2 L127N
external pH 6.5
internal pH 6.2
TASK-2 L127N
external pH 6.5
internal pH 7.5
TASK-2 L127N
external pH 6.5
internal pH 9.5
patch 1
patch 2
patch 3
Events
Open Events
Current (pA)
18.8 s
Po = 0.06
47.4 s
Po = 0.21
20.86 s
Po = 0.22
72.6 s
Po = 0.014
154.1 s
Po = 0.011
70.2 s
Po = 0.26
60.2 s
Po = 0.008
60.2 s
Po = 0.012
60.3 s
Po = 022

D

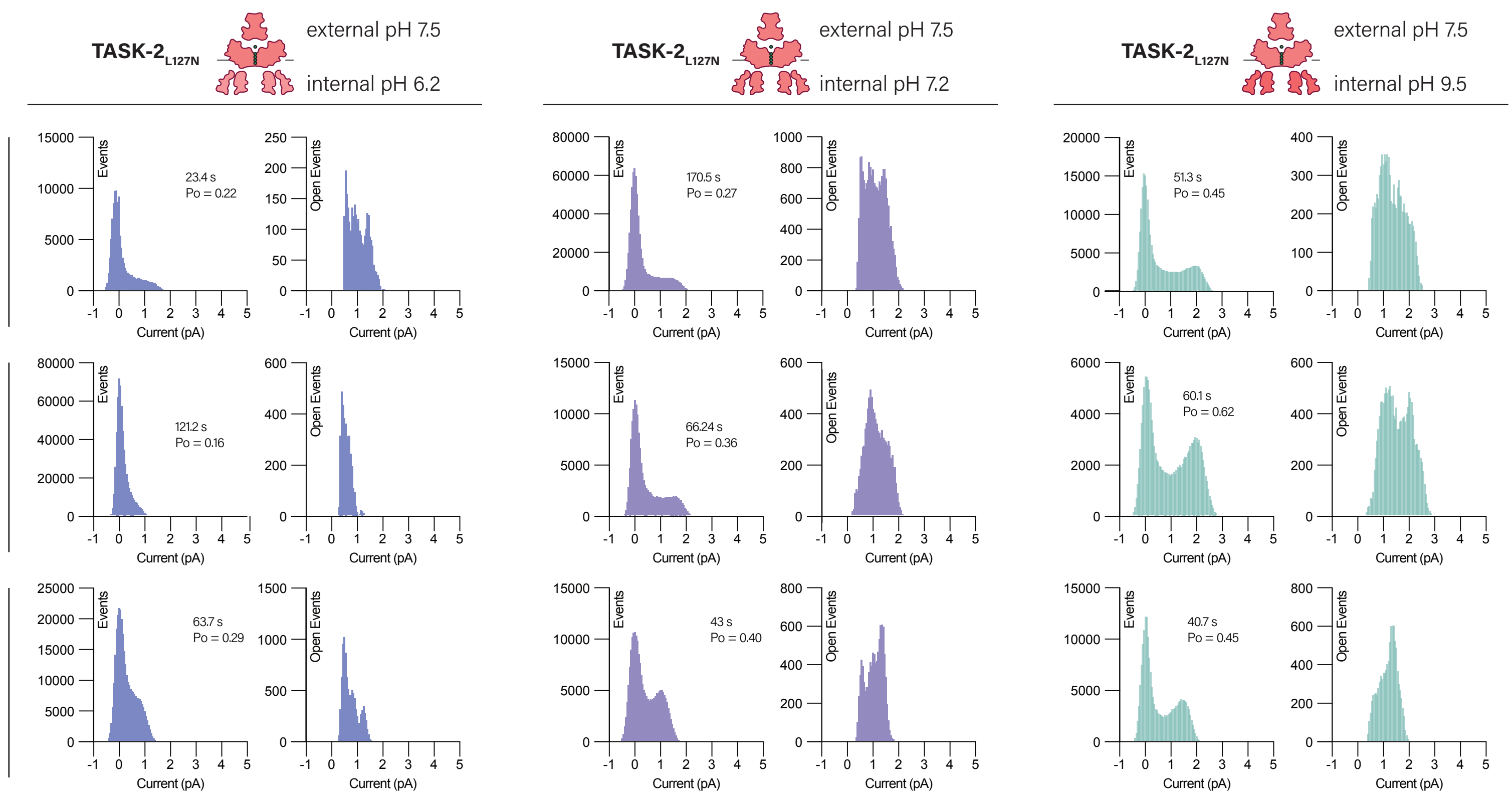
TASK-2 L127N
external pH 7.5
internal pH 6.2
TASK-2 L127N
external pH 7.5
internal pH 7.2
TASK-2 L127N
external pH 7.5
internal pH 9.5
patch 1
patch 2
patch 3
Events
Open Events
Current (pA)
23.4 s
Po = 0.22
170.5 s
Po = 0.27
51.3 s
Po = 0.45
121.2 s
Po = 0.16
66.24 s
Po = 0.36
60.1 s
Po = 0.62
63.7 s
Po = 0.29
43 s
Po = 0.40
40.7 s
Po = 0.45

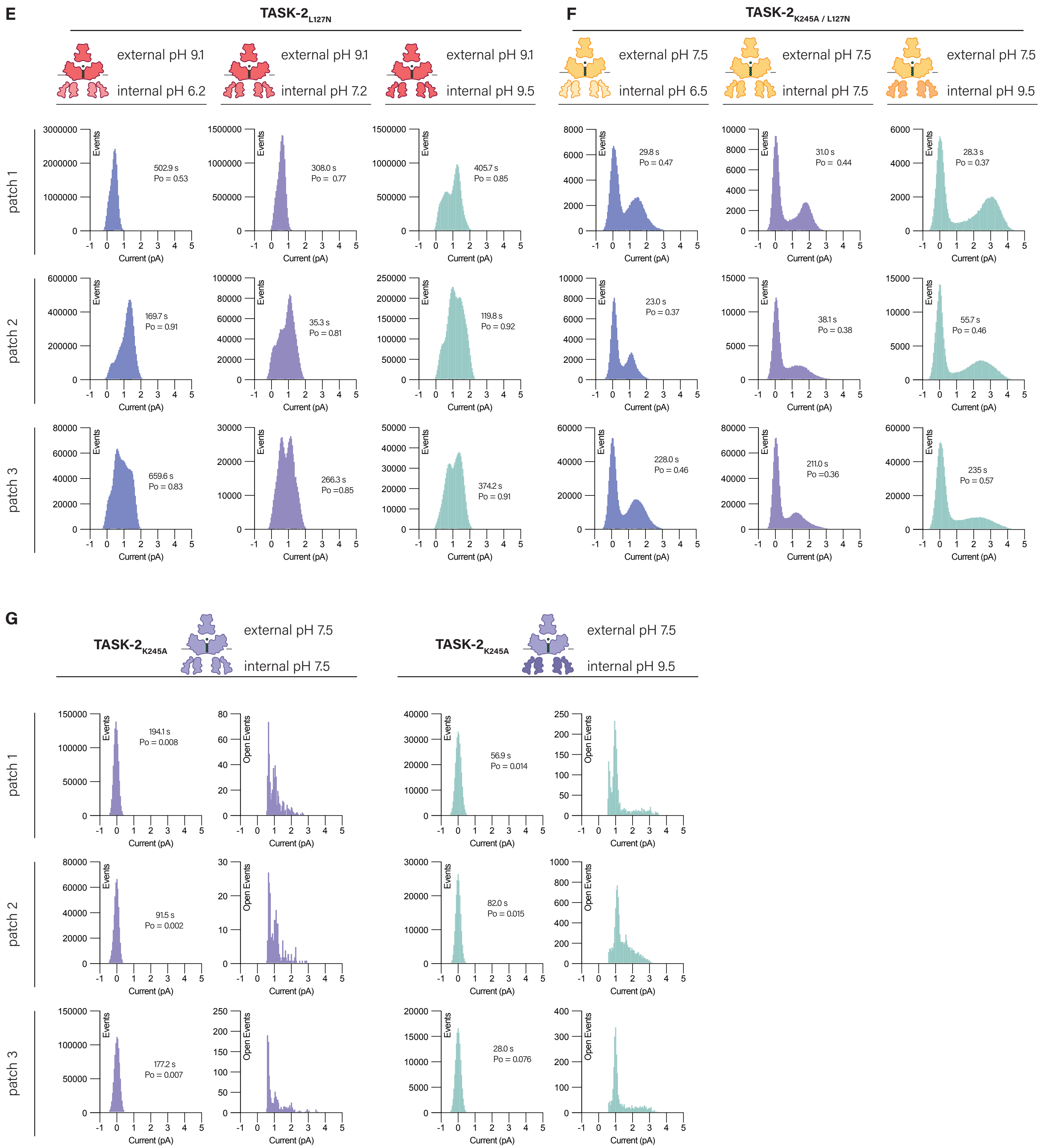

Figure S12. Single channel amplitude histograms.
Amplitude histograms from single channel recordings. For recordings in which channel open probability was low, all event (left) and open event (right) histograms are shown. Constructs and conditions are indicated above histograms. Duration of each record and calculated open probability (P_o) are indicated on all event histograms. Each patch was subjected to multiple pHint values as shown in rows. **(A)** TASK-2_{WT} pH_{ext} 7.5, **(B)** TASK-2_{WT} pH_{ext} 9.1, **(C)** TASK-2_{L127N} pH_{ext} 6.5, **(D)** TASK-2_{L127N} pH_{ext} 7.5, **(E)** TASK-2_{L127N} pH_{ext} 9.1, **(F)** TASK-$2_{L127N/K245A}$ pH_{ext} 7.5, and **(G)** TASK-2_{K245A} pH_{ext} 7.5.

SI References

1. Li, B., Rietmeijer, R. A. & Brohawn, S. G. Structural basis for pH gating of the two-pore domain K+ channel TASK2. *Nature* 586, 457–462 (2020).
2. Scheres, S. Processing of structurally heterogeneous cryo-EM data in RELION. *Methods Enzymol.* 579, 125–157 (2016).
3. Zivanov, J., Nakane, T. & Scheres, S. H. W. Estimation of high-order aberrations and anisotropic magnification from cryo-EM data sets in RELION-3.1. *IUCrJ* 7, 253–267 (2020).
4. Bepler, T. *et al.* Positive-unlabeled convolutional neural networks for particle picking in cryo-electron micrographs. *Nat. Methods* 16, 1153–1160 (2019).
5. Punjani, A., Rubinstein, J. L., Fleet, D. J. & Brubaker, M. A. cryoSPARC: algorithms for rapid unsupervised cryo-EM structure determination. *Nat. Methods* 14, 290–296 (2017).
6. Afonine, P. V. *et al.* Real-space refinement in PHENIX for cryo-EM and crystallography. *Acta Crystallogr. D Struct. Biol.* 74, 531–544 (2018).
7. Emsley, P., Lohkamp, B., Scott, W. G. & Cowtan, K. Features and development of coot. *Acta Crystallogr. D Biol. Crystallogr.* 66, 486–501 (2010).
8. Liebschner, D. *et al.* Macromolecular structure determination using X-rays, neutrons and electrons: recent developments in Phenix. *Acta Crystallogr. D Struct. Biol.* 75, 861–877 (2019).
9. Williams, C. J. *et al.* MolProbity: More and better reference data for improved all-atom structure validation. *Protein Sci.* 27, 293–315 (2018).
10. Sorum, B., Czégé, D. & Csanády, L. Timing of CFTR pore opening and structure of its transition state. *Cell* 163, 724–733 (2015).
